# Cardiac protein changes in ischaemic and dilated cardiomyopathy: a proteomic study of human left ventricular tissue

**DOI:** 10.1111/j.1582-4934.2012.01565.x

**Published:** 2012-09-26

**Authors:** Esther Roselló-Lletí, Jana Alonso, Raquel Cortés, Luis Almenar, Luis Martínez-Dolz, Ignacio Sánchez-Lázaro, Francisca Lago, Inmaculada Azorín, Jose R González Juanatey, Manuel Portolés, Miguel Rivera

**Affiliations:** a Cardiocirculatory Unit Research Center, Hospital Universitario La FeValencia, Spain; b Proteomics Laboratory Instituto de Investigación Sanitaria de Santiago, Hospital Clínico UniversitarioSantiago de Compostela, Spain; c Cardiology Unit, Hospital Universitario la FeValencia, Spain; d Research Laboratory 7 (Molecular and Cellular Cardiology), Santiago University Clinical HospitalSantiago de Compostela, Spain; e Experimental Neurology Research Center, Hospital Universitario La FeValencia, Spain; f Cardiology Unit, Hospital ClínicoSantiago de Compostela; g Cell Biology and Pathology Unit Research Center, Hospital Universitario La FeValencia, Spain

**Keywords:** proteomics, heart failure, ischaemic, dilated

## Abstract

The development of heart failure (HF) is characterized by progressive alteration of left ventricle structure and function. Previous works on proteomic analysis in cardiac tissue from patients with HF remain scant. The purpose of our study was to use a proteomic approach to investigate variations in protein expression of left ventricle tissue from patients with ischaemic (ICM) and dilated cardiomyopathy (DCM). Twenty-four explanted human hearts, 12 from patients with ICM and 12 with DCM undergoing cardiac transplantation and six non-diseased donor hearts (CNT) were analysed by 2DE. Proteins of interest were identified by mass spectrometry and validated by Western blotting and immunofluorescence. We encountered 35 differentially regulated spots in the comparison CNT *versus* ICM, 33 in CNT *versus* DCM, and 34 in ICM *versus* DCM. We identified glyceraldehyde 3-phophate dehydrogenase up-regulation in both ICM and DCM, and alpha-crystallin B down-regulation in both ICM and DCM. Heat shock 70 protein 1 was up-regulated only in ICM. Ten of the eleven differentially regulated proteins common to both aetiologies are interconnected as a part of a same network. In summary, we have shown by proteomics analysis that HF is associated with changes in proteins involved in the cellular stress response, respiratory chain and cardiac metabolism. Although we found altered expression of eleven proteins common to both ischaemic and dilated aetiology, we also observed different proteins altered in both groups. Furthermore, we obtained that seven of these eleven proteins are involved in cell death and apoptosis processes, and therefore in HF progression.

## Introduction

Heart failure (HF) is a progressive disorder characterized by poor prognosis and quality of life. It constitutes a major public health burden in the western world. This complex syndrome consists of not only haemodynamic abnormalities, but also cellular and molecular alterations in cardiac tissue. The development of chronic HF is characterized by progressive alteration of left ventricle structure and function, but the mechanism of these changes is still unclear and remains under intense study. Proteomic studies can serve as a preliminar approach of focused biochemical and physiological design to confirm changes associated not only with cardiovascular dysfunction but also with pharmacological interventions taken in response to this dysfunction.

To date, most studies on HF in human cardiomyocytes have focused on alterations in specific candidate proteins that have an important role in its physiopathology [1–3]. Moreover, differential proteomic approach has been applied to the search for novel proteins involved in the diseased heart, providing a new insight into the cellular mechanisms implied in cardiac dysfunction, and, furthermore offer the possibility of understanding the function of the cardiovascular system in all of its complexity [4–9]. In recent years, several studies based on the proteomic analysis of the left ventricle in animal models of heart disease or HF have shown changes in proteins involved in cardiac metabolism, associated with mitochondria and energy production, stress responses and structural proteins [10–13]. However, previous works on the proteomic analysis of left ventricular tissue from patients with ischaemic and dilated cardiomyopathy remain scant [7–9]. In fact, there is one recent proteomic study performed on human left ventricular tissue, but only from patients with DCM [9].

We hypothesize that patients with HF present different changes in left ventricular protein expression involved in essential cellular mechanisms according to the aetiologic diagnosis. The purpose of our study was therefore to use a proteomic approach to investigate variations in protein expression of left ventricle tissue from patients with ICM and DCM. We identified several proteins involved in the mitochondrial respiratory chain, associated with stress response, related with cellular metabolism and structural proteins, as potential cellular biomarkers for HF. We focused on three proteins with different expression between control and diseased, and found that glyceraldehyde 3-phophate dehydrogenase (G3P), heat shock 70 protein 1 (HSP71) and alpha-crystallin B (CRYAB) could have an important role in these cardiomyopathies.

## Materials and methods

### Collection of samples

Experimental material was taken from 24 explanted human hearts, 12 from patients with ICM and 12 with DCM undergoing cardiac transplantation. Clinical history, haemodynamic studies, electrocardiogram, Doppler echocardiography and coronary angiography data were available on all these patients. All patients were functionally classified according to the New York Heart Association (NYHA) criteria and were receiving medical treatment following the guidelines of the European Society of Cardiology [14], with diuretics 92%, aldosterone antagonists 83%, β-blockers 75%, angiotensin-converting enzyme inhibitors 68%, digoxin 50% and statins 42%. Non-ischaemic DCM was diagnosed when patients had intact coronary arteries on coronary angiography and left ventricular systolic dysfunction (ejection fraction <40%) with a dilated non-hypertrophic left ventricle (left ventricular diastolic diameter >55 mm) on echocardiography. We have chosen patients with DCM without family history of this pathology. Furthermore, patients did not show existence of primary valvular disease and no patient was treated by direct current shock before heart explantation.

Six non-diseased donor hearts were used as control (CNT) samples. All donors had normal left ventricular function and no history of myocardial disease or active infection at the time of transplantation. The hearts were initially considered for cardiac transplantation but were subsequently deemed unsuitable for transplantation either because of blood type or size incompatibility. The cause of death was cerebrovascular accident or motor vehicle accident.

Transmural samples were taken from near the apex of the left ventricle (in the ischaemic patients we used the peri-infarct zone), maintained in 0.9% NaCl throughout the extraction procedure and stored at 4°C for a maximum of 6 hrs from the time of coronary circulation loss. All tissues were obtained with informed consent of patients.

All patients gave written informed consent to participate in the study. The project was approved by the local Ethics Committe (Biomedical Investigation Ethics Committe of “La Fe “Universitary Hospital of Valencia, Spain) and the research was carried out according to the World Medical Association Declaration of Helsinki.

### Tissue homogenization

Frozen ventricular tissue was homogenized using a mortar and a pestle, in 400 μl lysis buffer [50 mM Tris-HCl (pH 7.4), 0.5 mM ethylenediaminetetraacetic acid, 1.25 mM dithiothreitol (DTT) and 0.5% Tween 20] supplemented with 4 μl phosphatase inhibitor cocktail (Sigma-Aldrich, Madrid, Spain) and 4 μl protease inhibitor cocktail (Sigma-Aldrich). The homogenate was sonicated twice, with 5 sec pulses (Sonifier 150, Branson, Emerson, MO, USA). Then, it was centrifuged at 10,000 g for 10 min. and the pellet was discarded. For protein extraction, 100 μl of supernatant was subjected to precipitation [2-D Clean-Up kit, GE Healthcare (Uppsala, Sweden)] according to the manufacturer's protocol. The resulting protein pellet was then resuspended in 50 μl 2D sample buffer [5 M urea, 2 M thiourea, 2 mM tributyl-phosphine, 65 mM DTT, 4% (w/v) 3-[(3-cholamidopropyl)dimethylammonio]-1-propanesulfonate (CHAPS), 1.6% (w/v) dimethylbenzylammonium propane sulfonate-256 (NDSB-256)] and stored at −20 °C. The protocol followed was applied after comparing the different protein extraction methods which involved different homogenization techniques (data not shown), making sure that the resulting pellet only included non-homogenate tissue.

### Two-dimensional gel electrophoresis

Protein quantitation was carried out with the Coomassie Plus protein reagent (Thermo Scientific, Asheville, NC, USA). For each sample, 300 μg of protein was resuspended in 300 μl of 2D sample buffer. Before passive rehydration of immobilized pH gradient (IPG) strips (3–10 Non-Linear, 18 cm, GE Healthcare), ampholytes were added to the sample at 0.1% Servalyte 3–10, 0.05% Servalyte 2–4 and 9–11 (SERVA, Germany). Isoelectric focusing was achieved in a Protean IEF cell (BioRad, CA, USA) following the manufacturer's protocol. Succeeding focusing, the IPG strips were immediately equilibrated for 10 min. in equilibration buffer (4 M urea, 2 mM thiourea, 12 mM DTT, 50 mM Tris pH 6.8, 2% SDS, 30% glycerol). The IPG strips were placed on top of the second dimension gels and embedded with 1% melted agarose. Proteins were separated in the second dimension by SDS-PAGE on 12.5% gels at running conditions of 18°C, 20 mA per gel for 16 hrs using Proteome Plus Dodeca cell (BioRad). Following electrophoresis, gels were fixed in 10% methanol/7% acetic acid for 1 hr, and stained 16 hrs with Sypro Ruby fluorescent dye (Lonza, Switzerland). After staining, gels were washed for 1 hr in 10% methanol/7% acetic acid and scanned in a Typhoon 9410 (GE Healthcare).

### Differential image analysis

The scanned gel images corresponding to CNT, ICM and DCM samples were sent to the Ludesi Analysis Center (Lund, Sweden, http://www.ludesi.com) for professional image analysis performed with Ludesi REDFIN 3 software. Spot detection, segmentation and matching followed a strict protocol to ensure a high level of correctness. Gels were matched using all-to-all spot matching, avoiding the bias caused by the use of a reference gel. Manual editing of spots was performed when needed. The integrated intensity of each of the spots was measured, and the background corrected and normalized. Spot volumes were normalized to the sum of all spot volumes in the gel image. Normalization removes systematic gel intensity differences originating, for example, from variations in staining, scanning time and protein loading by mathematically minimizing the median expression difference between matched spots. This allows a satisfactory quantification and comparison of different gels. Differential expression of spots was defined on the basis of >1.75-fold change between group averages and *P* < 0.05 [10]. Differences involving at least 94% of the samples of each group were considered meaningful.

### Mass spectrometric analysis

Spots chosen for mass spectrometric analysis were excised from the gels and manually in-gel digested with trypsin (Promega, Madison, WI, USA) as described previously [15]. Spots were reduced with DTT and alkylated with iodoacetamide prior to trypsin digestion. For mass spectrometry (MS) analysis, dried peptides were dissolved in 3 μl of 0.5% HCOOH. Equal volumes (0.5 μl) of peptide and matrix solution, consisting of 3 mg CHCA dissolved in 1 ml of 50% acetonitrile in 0.1% trifluoroacetic acid, were deposited using the thin layer method onto a 384 Opti-TOF (Time of Flight) MALDI (Matrix Assisted Laser Desorption/Ionization) plate (Applied Biosystems, CA, USA). Mass spectrometric data were obtained in an automated analysis loop using 4800 MALDI-TOF/TOF analyzer (Applied Biosystems). The MS spectra were acquired in reflectron positive-ion mode with a Nd:YAG, 355 nm wavelength laser, averaging 1000 laser shots and at least three trypsin autolysis peaks were used as internal calibration. All MSMS spectra were performed by selecting the precursors with a relative resolution of 300 (Full Width at Half Maximum) and metastable suppression. Automated analysis of mass data was achieved using the 4000 Series Explorer software v3.5.The MS and MSMS spectra data were combined through the Global Proteome Server Explorer software v3.6 using Mascot software v2.1 (Matrix Science) to search against a non-redundant database (SwissProt release 56.0), with 30 p.p.m. precursor tolerance, 0.35 Da MSMS fragment tolerance and allowing 1 missed cleavage. Protein scores greater than 56 were accepted as significant (*P* < 0.05), considering positive the identification whose protein score Confidence Interval % (CI) was above 98. In the case of MSMS spectra, total ion score CI% was above 94.

### Western blotting analysis

For 2D Western blot analysis, 80 μg of protein was resuspended in 120 μl of 2D sample buffer and isoelectrofocusing were performed in IPG strips 3–10 Non-Linear, 7 cm (BioRad), following the manufacturer's protocol. As described previously, IPG strips were placed on 12% SDS polyacrylamide gel and electrotransferred onto nitrocellulose membranes (HyBond, GE Healthcare). The primary detection antibodies used were: anti-GAPDH monoclonal antibody (1:10000) and anti-HSPA1B monoclonal antibody (1:15000), from AbCam (Cambridge, UK), heretofore blocking them in 5% bovine serum albumin (BSA) in Tris Buffered Saline (TBS-T). Membranes were exposed to horseradish peroxidase-labelled goat antimouse antibody (1:2000) (Santa Cruz Biotechnology, Inc., Delaware, CA, USA) and processed using an enhanced chemiluminiscence system (Immobilon Western, Millipore Corporation, MA, USA).

### Fluorescence microscopy

Frozen muscular sections were transferred to glass slides, fixed in 4% paraformaldehyde for 15 min. at 4°C. Then samples were blocked with PBS containing 1% BSA for 15 min. at room temperature. After blocking, sections were incubated for 120 min. at room temperature with the primary antibodies (described in western blot analysis) in the same buffer solution, and then with fluorescein isothiocyanate (FITC)-conjugated secondary antibody (Santa Cruz Biotechnology INC) for 60 min. at room temperature [16]. Finally, sections were rinsed in PBS, mounted in Vectashield conjugated 4′,6-diamidino-2-phenylindole (DAPI) for identifying nucleous (Vector Laboratories, CA, UK), and observed with an Olympus BX50 fluorescence microscope (Tokyo, Japan). The images were processed with ImageJ (v. 1.4.3.67) program.

### Ingenuity Pathways Analysis

Ingenuity Pathways Analysis software (Ingenuity Systems, CA, USA) was used to investigate possible interactions between all the identified proteins common in both aetiologies. Interactive pathways were generated to observe potential direct and indirect relations among the differentially expressed proteins.

### Statistical methods

Data are presented as the mean value ± standard deviation. The Kolmogorov-Smirnov test was used to analyse the distribution of the variables. Comparisons of clinical characteristics were achieved using Student's *t-*test for continuous variables and Fisher exact test for discrete variables. Comparison of protein expression levels between groups was performed with the normalized spot volumes and applying the Mann–Whitney *U-*test. Significance was assumed as *P* < 0.05. All statistical analyses were performed with SPSS software v. 15 for Windows (SPSS Inc.).

## Results

### Clinical characteristics of patients

All patients were men with a mean age of 49 ± 10 yrs. These patients had a NYHA functional classification of III–IV and were previously diagnosed with significant comorbidities including hypertension, hypercholesterolemia, obesity and diabetes mellitus. The clinical and echocardiographic characteristics of patients according to HF aetiology are summarized in Table 1. The DCM group was significantly younger compared with ICM patients (*P* < 0.01). Significant differences were also found in left ventricular end systolic diameter (*P* < 0.0001), left ventricular end diastolic diameter (*P* < 0.0001), left ventricular mass (*P* < 0.01) and left ventricular mass index (*P* < 0.01) as an increase in the DCM compared with ICM group. No statistically significant changes were found in the different drugs administered according to aetiology of HF, except for statins (*P* < 0.05). Six non-diseased donor hearts were used as control (CNT) samples (67% male, mean age 49 ± 16 yrs, ejection fraction >50%).

**Table 1:** Clinical and echocardiographic characteristics according to heart failure aetiology

	ICM(*n*= 12)	DCM(*n*= 12)
Age (years)	55 ± 7	44 ± 10†
Gender male (%)	100	100
BMI (kg/m^2^)	28 ± 3	27 ± 4
Prior hypertension (%)	45	25
Prior smoking (%)	100	58*
Diabetes mellitus (%)	64	0†
NYHA class	3.6 ± 0.4	3.5 ± 0.4
Haemoglobin (mg/dl)	14 ± 2	13 ± 1
Haematocrit (%)	42 ± 5	41 ± 5
Total cholesterol (mg/dl)	161 ± 41	142 ± 41*
Duration of disease (months)	57 ± 44	52 ± 43
Number of myocardial infarctions	1.5 ± 0.7	0*
Echo-Doppler study		
Ejection fraction (%)	23 ± 4	19 ± 7
Fractional shortening (%)	13 ± 4	10 ± 4
Left ventricular end systolic diameter (mm)	55 ± 9	72 ± 7‡
Left ventricular end diastolic diameter (mm)	64 ± 9	81 ± 8‡
Left ventricle mass (g)	253 ± 69	403 ± 126†
Left ventricle mass index (g/cm^2^)	133 ± 30	201 ± 48†
Treatment (%)		
Diuretics	92	92
Aldosterone antagonists	75	92
β-blockers	58	92
Angiotensin-converting enzyme inhibitors	67	67
Digoxin	50	50
Statins	67	17*

Duration of disease from diagnosis of heart failure until heart transplant.

BMI: body mass index; DCM: dilated cardiomyopathy; NYHA: New York Heart Association.

* *P* < 0.05;

† *P* < 0.01;

‡ *P* < 0.0001.

### Proteome analysis of left ventricular tissue by 2D electrophoresis

We performed 2D gel electrophoresis (2DE) to expand our knowledge on the proteome changes associated with ICM and DCM. The proteome analysis was carried out on left ventricular tissue of patients with HF. After the optimization of the experimental conditions for tissue homogenization, protein extraction and separation by 2DE, we standardized experimental conditions resulting in reproducible 2D gels (18 × 19 cm) for 3–10 NL pH gradient. We detected a mean of 876 ± 56.7 spots corresponding to left ventricular tissue from CNT, 900 ± 43.2 from ICM and 895 ± 39.7 from DCM.

**Fig 1:**
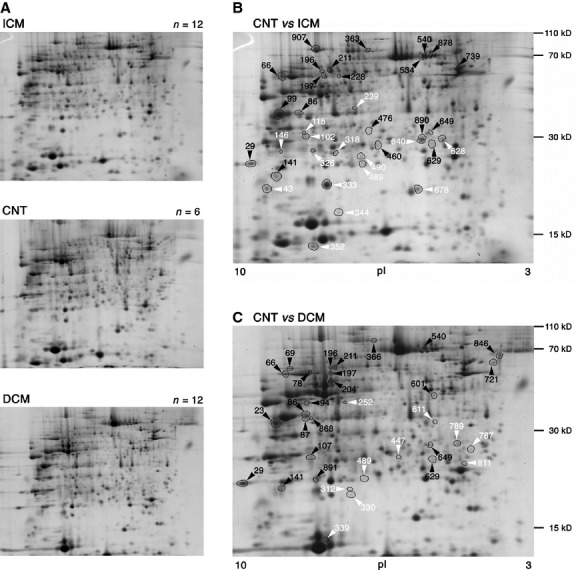
2-DE-based proteome analysis of LV tissue from patients with ICM and DCM. Representative 2-DE image of LV tissue proteins of ICM, DCM and CNT samples (3–10 pH range, 12.5% SDS polyacrylamide gel in the second dimension) (A). Black arrowheads indicate spots with higher expression levels, whereas white arrowheads designate under-expression spots. In both cases compared with CNT (B, C). CNT: control; DCM: dilated cardiomyopathy; ICM: ischaemic cardiomyopathy; LV: left ventricular.

Thirty gels, six from CNT samples, and 12 for each cardiomyopathy (12 ICM and 12 DCM) were submitted to differential image analysis. We focused on the identification of up- and down-regulation of spot intensities where the fold change was at least 1.75 (with *P* < 0.05). Furthermore, for the analysis we only considered spots that were present in at least 94% of the gels in a given group (CNT, ICM or DCM). These conservative criteria for protein detection aimed to avoid misidentifications due to gel-to-gel variation. Taking into account these conservative criteria, we encountered 35 differentially regulated spots in the comparison of CNT *versus* ICM (Fig. 1A), 33 in CNT *versus* DCM (Fig. 1B), and 34 in ICM *versus* DCM. All were successfully identified by MS (Tables S1, S2 and S3, respectively).

In the case of the 35 ICM spots identified, 15 protein features were down-regulated, while 20 were up-regulated. They correspond to 33 open reading frames (ORFs), with four ORFs being represented by two spots (86 and 99; 102 and 115; 196 and 211; 489 and 490). The 33 spots with differential expression in DCM correspond to 31 ORFs, with three ORFs being represented by two spots (66 and 69; 196 and 211; 789 and 811) and one ORF represented by three spots (86, 87 and 868). Nine spots were down-regulated in DCM gels, whereas 24 were up-regulated. We identified spots with more than one protein, concretely, spots 196 and 344 in ICM (Table 2) and spots 204, 339 and 366 in DCM (Table 3), as a result of coincidence in pI and molecular weight.

**Table 2:** Ventricle proteins differentially regulated in CNT *versus* ICM patients

Spot	Protein	Accesion number	Fold-change	*P*-value	Function
29	**ATP synthase subunit O, mitochondrial**	ATPO_HUMAN	+3.20	0.0048	Respiratory chain
43	NADH dehydrogenase [ubiquinone] iron-sulphur protein 4, mitochondrial	NDUS4_HUMAN	−1.81	7.630e−4	Respiratory chain
66	Ig gamma-1 chain C region	IGHG1_HUMAN	+3.48	0.0048	Immune response
86	**Glyceraldehyde-3-phosphate dehydrogenase**	G3P_HUMAN	+2.53	1.077e−4	Metabolism
99	**Glyceraldehyde-3-phosphate dehydrogenase**	G3P_HUMAN	+1.97	4.259e−4	Metabolism
102	Adenylate kinase 2, mitochondrial	KAD2_HUMAN	−1.93	0.0021	Metabolism
115	Adenylate kinase 2, mitochondrial	KAD2_HUMAN	−3.75	4.259e−4	Metabolism
141	**NADH dehydrogenase [ubiquinone] 1 beta subcomplex 9**	NDUB9_HUMAN	+3.30	0.0021	Respiratory chain
146	Protein NipSnap homolog 3A	NPS3A_HUMAN	−2.59	0.0134	Miscellaneous
196	Electron transfer flavoprotein-ubiquinone oxidoreductase, mitochondrial	ETFD_HUMAN	+2.27	0.0097	Transport
	**Dihydrolipoyl dehydrognease, mitochondrial**	DLDH_HUMAN			Metabolism
197	**ATP synthase subunit alpha, mitochondrial**	ATPA_HUMAN	+5.19	1.077e−4	Respiratory chain
211	**Dihydrolipoyl dehydrogenase, mitochondrial**	DLDH_HUMAN	+2.92	1.077e−4	Metabolism
228	Succinyl-CoA:3-ketoacid-coenzyme A transferase 1, mitochondrial	SCOT1_HUMAN	+4.35	2.173e−4	Metabolism
239	Malate dehydrogenase, cytoplasmic	MDHC_HUMAN	−2.31	4.259e−4	Metabolism
318	ES1 protein homolog, mitochondrial	ES1_HUMAN	−2.37	0.0182	Miscellaneous
326	3-hydroxyacyl-Coa dehydrogenase type-2	HCD2_HUMAN	−3.11	2.173e−4	Metabolism
333	**Alpha-crystallin B chain**	CRYAB_HUMAN	−1.98	0.0321	Structural
344	Myoglobin	MYG_HUMAN	−2.48	1.077e−4	Transport
	**Peptidyl-prolyl cis-trans isomerase A**	PPIA_HUMAN			Immune response
352	D-dopachrome decarboxylase	DOPD_HUMAN	−2.28	0.0021	Metabolism
363	**Ezrin**	EZRI_HUMAN	+3.05	1.077e−4	Structural
460	Ig kappa chain C region	IGKC_HUMAN	+2.18	0.0244	Immune response
476	Cytochrome c1, haem protein, mitochondrial	CY1_HUMAN	+3.31	0.0097	Respiratory chain
489	Cytochrome b-c1 complex subunit Rieske, mitochondrial	UCR1_HUMAN	−1.79	0.0244	Respiratory chain
490	**Cytochrome b-c1 complex subunit Rieske, mitochondrial**	UCR1_HUMAN	−1.91	7.630e−4	Respiratory chain
534	Heat shock 70 kD protein 1	HSP71_HUMAN	+3.31	4.259e−4	Stress response
540	**Stress-70 protein, mitochondrial**	GRP75_HUMAN	+1.64	1.077e−4	Stress response
628	Glutathione S-transferase Mu 3	GSTM3_HUMAN	−2.29	4.259e−4	Metabolism
629	Peroxiredoxin-2	PRDX2_HUMAN	+2.96	0.0048	Stress response
640	Heat shock protein beta-1	HSPB1_HUMAN	−2.39	4.259e−4	Stress response
649	**Prohibitin**	PHB_HUMAN	+2.25	2.173e−4	Miscellaneous
678	Heat shock protein beta-6	HSPB6_HUMAN	−1.76	2.173e−4	Stress response
739	Alpha-1-antitrypsin	A1AT_HUMAN	+2.43	1.077e−4	Miscellaneous
878	Heat shock cognate 71 kD protein	HSP7C_HUMAN	+2.61	0.0048	Stress response
890	NADH dehydrogenase [ubiquinone] iron-sulphur protein 3, mitochodrial	NDUS3_HUMAN	+2.51	0.0097	Respiratory chain
907	Aconitase hydratase, mitochondrial	ACON_HUMAN	+3.92	4.259e−4	Metabolism

A negative-fold change indicates that the protein feature is down-regulated in ICM whereas a positive-fold change indicates the spot is up-regulated in this pathological group. The proteins shown in bold are altered in both ICM and DCM aetiology.

CNT: control; DCM: dilated cardiomyopathy; ICM: ischaemic cardiomyopathy.

**Table 3:** Ventricle proteins differentially regulated in CNT *versus* DCM patients

Spot	Protein	Accesion number	Fold-change	*P*-value	Function
23	ATP synthase subunit G, mitochondrial	ATPG_HUMAN	+3.71	1.077e−4	Respiratory chain
29	**ATP synthase subunit O, mitochondrial**	ATPO_HUMAN	+3.79	7.630e−4	Respiratory chain
66	**ATP synthase subunit A, mitochondrial**	ATPA_HUMAN	+3.25	1.077e−4	Respiratory chain
69	**ATP synthase subunit A, mitochondrial**	ATPA_HUMAN	+2.31	0.0069	Respiratory chain
78	UTP-glucose-1-phosphate urydylyltransferase	UGPA_HUMAN	+3.19	1.077e−4	Metabolism
86	Annexin A2	ANXA2_HUMAN	+2.19	4.259e−4	Transport
87	Annexin A2	ANXA2_HUMAN	+3.90	1.077e−4	Transport
94	Acetyl-CoA acetyltransferase, mitochondrial	THIL_HUMAN	+2.30	0.0097	Metabolism
107	**Glyceraldehyde-3-phosphate dehydrogenase**	G3P_HUMAN	+2.89	2.173e−4	Metabolism
141	**NADH dehydrogenase [ubiquinone] 1 beta subcomplex subunit 9**	NDUB9_HUMAN	+2.23	0.0021	Respiratory chain
196	**Dihydrolipoyl dehydrogenase, mitochondrial**	DLDH_HUMAN	+2.25	0.0097	Metabolism
197	Glutamate dehydrogenase 1, mitochondrial	DHE3_HUMAN	+2.91	7.630e−4	Metabolism
204	Aspartate aminotransferase, cytoplasmic	AATC_HUMAN	+1.99	2.173e−4	Metabolism
	Alpha-enolase	ENOA_HUMAN			Metabolism
211	**Dihydrolipoyl dehydrogenase, mitochondrial**	DLDH_HUMAN	+2.10	0.0048	Metabolism
252	Alcohol dehydrogenase [NADP+]	AK1A1_HUMAN	−4.74	1.077e−4	Metabolism
312	**Alpha-crystallin B chain**	CRYAB_HUMAN	−2.10	0.0021	Structural
330	THAP domain-containing protein 4	THAP4_HUMAN	−1.92	0.0032	Miscellaneous
339	Peroxiredoxin-5	PRDX_HUMAN	−2.02	0.0021	Stress response
	**Peptidyl-prolyl cis-trans isomerase**	PPIA_HUMAN			Immune response
366	Mitochondrial inner membrane protein	IMMT_HUMAN	+3.13	0.0069	Miscellaneous
	**Ezrin**	EZRI_HUMAN			Structural
447	Metaxin-2	MTX2_HUMAN	−2.48	0.0097	Transport
489	**Cytochrome b-c1 complex subunit Rieske, mitochondrial**	UCR1_HUMAN	−2.66	4.259e−4	Respiratory chain
540	**Stress-70 protein, mitochondrial**	GRP75_HUMAN	+1.75	1.077e−4	Stress response
601	Stomatin-like protein 2	STML2_HUMAN	+2.02	0.0182	Structural
611	Glyoxalase domain-containing protein 4	GLOD4_HUMAN	−3.53	0.0013	Miscellaneous
629	Ubiquitin carboxyl-terminal hydrolase isozyme L1	UCHL1_HUMAN	+2.32	0.0097	Miscellaneous
649	**Prohibitin**	PHB_HUMAN	+1.93	0.0021	Miscellaneous
	Chloride intracellular channel protein 4	CLIC4_HUMAN			Transport
721	Alpha-2-HS-glycoprotein	FETUA_HUMAN	+3.38	0.0069	Miscellaneous
787	Calpain small subunit 1	CPNS1_HUMAN	−4.33	2.173e−4	Transport
789	Myosin light chain 3	MYL3_HUMAN	−2.28	0.0134	Structural
811	Myosin light chain 3	MYL3_HUMAN	−5.39	0.0021	Structural
846	Calreticulin	CALR_HUMAN	+2.14	0.0032	Stress response
868	Annexin A2	ANXA2_HUMAN	+2.25	4.259e−4	Transport
891	Flavin reductase	BLVRB_HUMAN	+3.34	0.0182	Metabolism

A negative-fold change indicates that the protein feature is down-regulated in DCM whereas a positive-fold change indicates the spot is up-regulated in this pathological group. The proteins shown in bold are altered in both ICM and DCM aetiology.

CNT: control; DCM: dilated cardiomyopathy; ICM: ischaemic cardiomyopathy.

The comparison between pathological groups (ICM and DCM) shows 34 differentially expressed spots, 14 up-regulated in ICM and 20 in DCM. They correspond to 33 ORFs, three of them represented by two spots (165 and 496; 326 and 460; 788 and 811), due to the existence of isoforms. We identified more than one protein in spots 18 and 788, as a consequence of their common pI and Mw.

In ICM, 82% of detected protein features showed a difference of not more than 6 kD compared with the theoretical values calculated on the basis of the ORFs. Inspection of pI showed that 74% of the spots had an experimental pI with no deviation over ± 0.5 from the theoretical value. In DCM, 76% exhibited a variation of not more than 8 kD in comparison with the theoretical values, and 61% had an experimental pI with a maximum deviation of ± 0.5. In the comparison between both pathologies, 52% showed a variation of not more than 8 kD in their Mw values with respect to their theoretical values, and 24% revealed an experimental pI deviation of ± 0.5, excluding Ig kappa chain C region with a shift of 15 kD, resulting in fragmented immunoglobulin. In the case of significant deviations in pI and Mw, we observed that they were a result of the presence of isoforms and/or posttranslational modifications, e.g. phosphorylation, glycations.

### Significant proteins identified

We show in Tables 2 and 3 that many of the proteins involved in cellular stress response, respiratory chain and cardiac metabolism are altered in human hearts with ICM or DCM. Some of these changes are common in both aetiologies, such as stress-70 protein mitochondrial, NADH dehydrogenase (ubiquinone) 1 beta subcomplex 9, dihydrolipoyl dehydrogenase or glyceraldehyde-3-phosphate dehydrogenase (G3P). However, most of the proteins altered in both groups with respect to control group do not match. Heat shock proteins (HSP71, HSPB1, HSPB6, HSP7C) are altered only in ICM group. The remaining proteins identified were basically structural or implicated in transport and immune response, such as alpha-crystallin B (CRYAB), myoglobin and peptidyl-prolyl cis-trans isomerase, respectively. When we compared the two pathological groups (ICM *versus* DCM), we identified new altered proteins that were not detected in the previous comparisons, e.g. metabolic enzymes or structural proteins, such as malate dehydrogenase or actin alpha cardiac muscle respectively (Table 4).

**Table 4:** Ventricle proteins differentially regulated in ICM *versus* DCM patients

Spot	Protein	Accesion number	Fold-change	*P*-value	Function
18	Malate dehydrogenase, mitochondrial	MDHM_HUMAN	2.41§	0.0068	Metabolism
	Succinyl-CoA ligase]GDP-forming] subunit alpha, mitochondrial	SUCA_HUMAN			Metabolism
70	Pyruvate kinase isozymes M1/M2	KPYM_HUMAN	2.93*	0.0014	Metabolism
78	**UTP-glucose-1-phosphatase uridylyltransferase**	UGPA_HUMAN	2.03*	0.0100	Metabolism
110	Galectin-3	LEG3_HUMAN	1.99‡	0.0014	Immune response
115	**Adenylate kinase 2, mitochondrial**	KAD2_HUMAN	4.64**	1.000e−4	Metabolism
121	2,4-dienoyl-CoA reductase, mitochondrial	DECR_HUMAN	2.83*	0.0036	Metabolism
165	Nucleoside diphosphate kinase B	NDKB_HUMAN	1.97†	1.000e−4	Metabolism
192	Alpha-enolase	ENOA_HUMAN	2.94†	0.0100	Metabolism
197	**ATP synthase subunit alpha, mitochondrial**	ATPA_HUMAN	1.78*	2.746e−4	Respiratory chain
228	**Succinyl-CoA:3-ketoacid-coenzyme A transferase 1, mitochondrial**	SCOT1_HUMAN	2.06*	0.0023	Metabolism
239	**Malate dehydrogenase, cytoplasmic**	MDHC_HUMAN	1.84†	3.712e−4	Metabolism
283	Ig lambda-3 chain C regions	LAC3_HUMAN	1.76‡	0.0045	Immune response
300	Voltage-dependent anion-selective channel protein 2	VDAC2_HUMAN	1.86†	0.0045	Transport
304	Immunoglobulin lambda-like polypeptide 5	IGLL5_HUMAN	2.23‡	0.0145	Immune response
318	**ES1 protein homolog, mitochondrial**	ES1_HUMAN	2.01†	0.0449	Miscellaneous
326	Ig kappa chain C region	IGKC_HUMAN	2.08†	0.0100	Immune response
335	Cofilin-2	COF2_HUMAN	2.42†	0.0068	Structural
344	**Myoglobin**	MYG_HUMAN	1.96†	1.000e−4	Transport
447	**Metaxin-2**	MTX2_HUMAN	2.23§	0.0242	Transport
460	**Ig kappa chain C region**	IGKC_HUMAN	3.26*	2.746e−4	Immune response
469	Delta(3,5)-Delta(2,4)-dienoyl-CoA isomerase,mitochondrial	ECH1_HUMAN	1.79§	0.0145	Metabolism
490	**Cytochrome b-c1 complex subunit Rieske, mitochondrial**	UCRI_HUMAN	1.92†	1.446e−4	Respiratory chain
496	Nucleoside diphosphate kinase A	NDKA_HUMAN	1.76‡	0.0011	Metabolism
553	Actin, alpha cardiac muscle	ACTC_HUMAN	2.40§	8.585e−4	Structural
601	**Stomatin-like protein 2**	STML2_HUMAN	1.97‡	0.0205	Sructural
611	**Glyoxalase domain-containing protein 4**	GLOD4_HUMAN	2.84§	0.0056	Miscellaneous
640	**Heat shock protein beta-1**	HSPB1_HUMAN	2.37†	1.000e−4	Stress response
739	**Alpha-1-antitrypsin**	A1AT_HUMAN	1.77*	1.000e−4	Miscellaneous
781	Tropomyosin alpha-4	TPM4_HUMAN	2.75‡	2.746e−4	Structural
788	Rho GDP-dissociation inhibitor 1	GDIR1_HUMAN	2.52†	0.0018	Signal Transduction
	Myosin light chain 4	MYL4_HUMAN			Structural
811	**Myosin light chain 3**	MYL3_HUMAN	3.67§	0.0083	Structural
812	Lactoylglutathione lyase	LGUL_HUMAN	1.87‡	0.0242	Metabolism
890	**NADH dehydrogenase [ubiquinone] iron-sulphur protein 3, mitochondrial**	NDUS3_HUMAN	2.99*	2.006e−4	Respiratory chain
1198	Heterogeneous nuclear ribonucleoproteins A2/B1	ROA2_HUMAN	3.49*	3.712e−4	Transport

Note

* Protein up-regulated in ICM,

† protein down-regulated in ICM,

‡ protein up-regulated in DCM,

§ protein down-regulated in DCM.

The number of fold-change is referred to the comparison between ICM and DCM. The direction of the fold-change values is referred to the protein levels of the CNT group. The proteins shown in bold were altered in any pathological group when we compared with CNT samples (Tables 2 and 3).

CNT: control; DCM: dilated cardiomyopathy; ICM: ischaemic cardiomyopathy.

**Fig 2:**
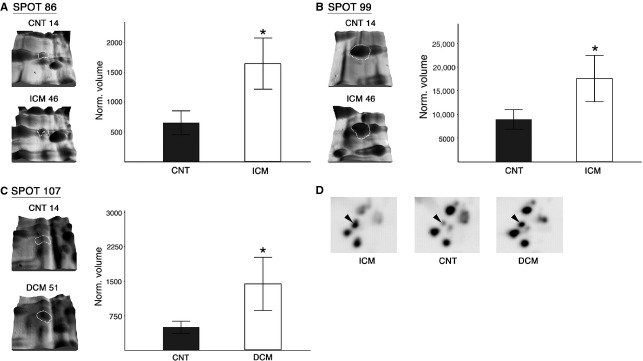
G3P is over-expressed in patients with ICM and DCM contrasted to CNT samples. The volume of spots 86 and 99 (white outline) in ICM (*n* = 12) shows a higher level of expression compared with CNT group (*n* = 6) (A, B). The volume of spot 107 (white outline) in DCM (*n* = 12) shows a higher level of expression compared with CNT (C). Representative image of 2D Western blot reveals an alteration in G3P expression (black arrowhead) between CNT and HF patients (D). Images are representative of the results obtained for all the patients included in the study. CNT: control; DCM: dilated cardiomyopathy; G3P: glyceraldehyde-3-phosphate dehydrogenase; ICM: ischaemic cardiomyopathy. *P < 0.0001.

**Fig 3:**
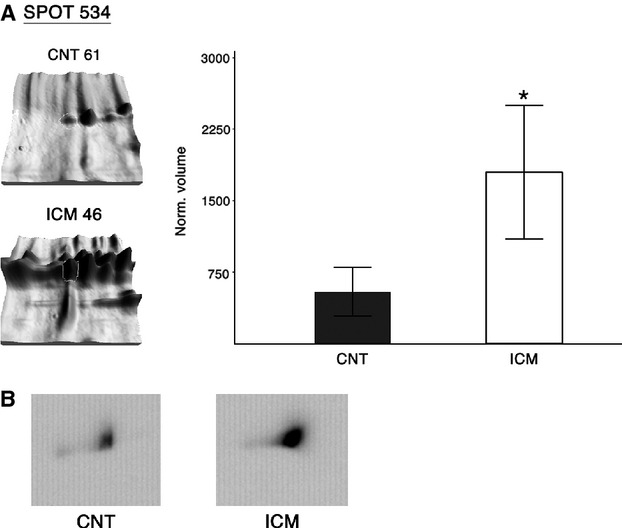
The HSP71 is over-expressed in patients with ICM contrasted to CNT samples. Spot 534 volumes (white outline) in CNT (*n* = 6) and patients with ICM (*n* = 12), exhibiting an expression increase in ischaemic samples compared with CNT (A). Representative image of 2D Western blot reveals an alteration in HSP71 expression between CNT and patients with ICM (B). Images are representative of the results obtained for all the patients included in the study. CNT: control; HSP71: heat shock 70 kD protein 1; ICM: ischaemic cardiomyopathy. *P < 0.0001.

A selection of the above proteins was validated by 2D western blotting. We identified G3P in the 2-DE analysis, being present in two very close spots with a pI difference of 0.56 units (see Fig. 1B, spots 86 and 99; 1C spot 107). These spots (Fig. 2A, 2B, 2C) were up-regulated in HF samples (ICM and DCM, Tables 2 and 3). The 2D Western blot analysis allows us the detection of changes in expression (Fig. 2D), showing alterations in a spot cluster expression. In addition, we identified heat shock 70 kD protein 1 (HSP71) in the 2-DE analysis only in the ischaemic group (Fig. 3A) and we validated the proteomic result by 2D Western blotting using a specific antibody, as shown in Figure 3B. The results were coincident with proteomic analysis.

**Fig 4:**
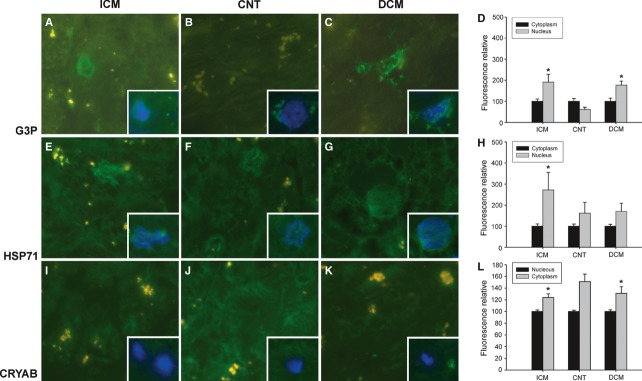
Effect of HF on cell distribution of G3P, HSP71 and CRYAB. Immunofluorescence for G3P (A–D), HSP71 (E–H) and CRYAB (I–L) according to HF aetiology, ischaemic (ICM) and dilated (DCM) cardiomyopathy compared with control group (CNT). Yellow spots are the fluorescence of lipofuscin particles. The insertions show the nucleus co-stained with DAPI (blue). All the micrographs are representative of the results obtained in four independent experiments for each group studied, ICM (*n* = 4), DCM (*n* = 4) and CNT (*n* = 4). The bar represents 10 μm. D, H, bar graph comparing the fluorescence intensity into nucleus of G3P and HSP71 in ischaemic and dilated compared with control hearts. The values from the cytoplasm were set to 100. The L, bar graph comparing the fluorescence intensity into cytoplasm of CRYAB in ischaemic and dilated compared with control hearts. The values from the nucleus were set to 100. The data are expressed as mean ± S.D. of four experiments. *P < 0.01. CRYAB: alpha-crystallin B; G3P: glyceraldehyde-3-phosphate dehydrogenase; HF: heart failure; HSP71: heat shock 70 kD protein 1.

Immunofluorescence studies showed that G3P intensity was higher in the nucleus than in the cytoplasm in the ischaemic and dilated hearts (91% and 77% respectively). Pathological hearts had an increase in nucleus intensity compared with control samples (*P* < 0.01). Furthermore, HSP71 was distributed on both the cytoplasm and nucleus in the three groups, but a significant higher percentage of fluorescence was measured inside the nucleus than outside (172% ischaemic, 62% controls, and 77% dilated hearts). Ischaemic group has an increase in nucleus intensity compared with control samples (*P* < 0.01). Finally, immunofluorescence analysis showed that CRYAB was distributed on cytoplasm and the intensity of this protein was lower in ischaemic and dilated hearts compared with control samples (*P* < 0.01). The results were also coincident with proteomic analysis (Fig. 4).

**Fig 5:**
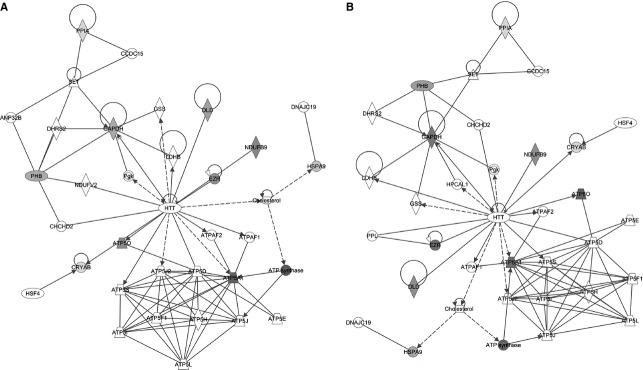
Analysis of differentially regulated proteins by Ingenuity Pathways Analysis software (Ingenuity Systems). Potential protein interactions according to HF aetiology, ischaemic (A) and dilated (B) cardiomyopathy are shown in following network: DNA replication, recombination, and repair, energy production, and nucleic acid metabolism. Proteins identified by differential analysis are shown as shaded nodes with their gene names. Solid lines represent direct interactions, dotted represent indirect interactions. Arrows from one node to another indicated that this node acts upon the other. Lines without arrows represent binding. Node shapes are: Double circle = complex or group, notched triangle = transporter, diamond = enzyme, oval = transcription regulator, circle = other. The ANP32B, Acidic leucine-rich nuclear phosphoprotein 32 family member B; ATP synthase subunit O, mitochondrial; ATP5A1, ATP synthase subunit alpha, mitochondrial; ATP5D, ATP synthase subunit delta, mitochondrial; ATP5E, ATP synthase subunit epsilon, mitochondrial; ATP5F1, ATP synthase subunit b, mitochondrial; ATP5H, ATP synthase subunit d, mitochondrial; ATP5I, ATP synthase subunit e, mitochondrial; ATP5J, ATP synthase-coupling factor 6, mitochondrial; ATP5J2, ATP synthase subunit f, mitochondrial; ATP5L, P synthase subunit g, mitochondrial; ATP5S, ATP synthase subunit s, mitochondrial; ATPAF1: ATP synthase mitochondrial F1 complex assembly factor 1; ATPAF2: ATP synthase mitochondrial F1 complex assembly factor 2;CCDC15: Coiled-coil domain-containing protein 15; CHCHD2: Coiled-coil-helix-coiled-coil-helix domain-containing protein 2: mitochondrial; CRYAB: Alpha-crystallin B chain; DHRS2: Dehydrogenase/reductase SDR family member 2; DLD: Dihydrolipoyl dehydrogenase: mitochondrial; DNAJC19: Mitochondrial import inner membrane translocase subunit TIM14; EZR: Ezrin; GADPH: Glyceraldehyde-3-phosphate dehydrogenase; GSS: Glutathione synthetase; HPCAL1: HPCAL1 protein; HSF4: Heat shock factor protein 4; HSPA9: Stress-70 protein: mitochondrial; HTT: Huntingtin; LDHB: L-lactate dehydrogenase B chain; LDHB: L-lactate dehydrogenase B chain; NDUFB9: NADH dehydrogenase [ubiquinone] 1 beta subcomplex subunit 9; NDUFV2: NDUFV2 protein; Pgk: Phosphoglycerate kinase Pgk; PHB: Prohibitin; PPIA: Peptidyl-prolyl cis-trans isomerase A; PPL: Ppl protein; SET: Protein SET.

Ingenuity Pathways Analysis software (Ingenuity Systems) was used to investigate possible interactions between the identified proteins common in both aetiologies to highlight predominant networks. Networks obtained in the two groups analysed were similar. Ten of the eleven differentially regulated proteins identified are interconnected as a part of a common network related to DNA replication, recombination and repair, energy production and nucleic acid metabolism (Fig. 5). Interestingly, seven of those proteins, besides presenting a known function in the cell, are also involved in cell death, specifically G3P, CRYAB, ATP synthase subunit alpha, ezrin, stress-70 protein, prohibitin and peptidyl-prolyl cis-trans isomerase A. All these proteins are related directly or indirectly with huntingtin and this calcium channel regulator plays an important role in cell death and apoptosis.

## Discussion

In the present study, we carried out a 2DE-based proteomic analysis of ventricular tissue from patients with ICM and DCM to investigate cardiac protein expression changes in HF, and to gain more knowledge on the cellular mechanisms related to the development of HF. Previous works on proteomic analysis in cardiac tissue from patients with HF are limited [4–9]. To date, proteomic investigations into heart disease have preferably focused on failing hearts due to DCM. In fact, to the best of our knowledge there are only two proteomic studies performed on human left ventricular tissue from patients with ICM and DCM [7, 8]. We want to emphasize the importance of having carried out this study in a significant number of samples from explanted human hearts from patients with ICM and DCM undergoing cardiac transplantation. This has allowed us to extract the region of tissue that we want to analyse, which could not have been possible if the study had been made with biopsies. In particular, our study showed a large number of proteins involved in cellular stress response, respiratory chain and cardiac metabolism that are altered in heart failure compared with the non-failing hearts.

We found 35 spots to be differentially regulated between left ventricular tissue from ICM and control group (20 increased, 15 decreased), and a total of 33 spots were altered in DCM (24 up-regulated, 9 down-regulated). We have shown that many of the underexpressed proteins in ICM are implicated in mitochondrial function, and it is known that mitochondrial dysfunction is involved in a variety of pathologies including heart disease and ischaemia/reperfusion [17]. We also found that some mitochondrial proteins increased in both aetiologies, concretely ATP synthase subunit O, ATP synthase subunit alpha, NADH dehydrogenase (ubiquinone) 1 alpha subcomplex subunit 10, dihydrolipoyl dehydrogenase, stress-70 protein and prohibitin. Mitochondria are a major source and target of free radicals and the collapse of the mitochondrial transmembrane potential can initiate the signaling cascades involved in apoptosis [18]. In addition, it plays an important role in the regeneration of antioxidants and it is responsible for the majority of ATP production [19]. Thus, characterization of the mitochondrial proteome could provide new insights into cardiac dysfunction in heart failure patients [17, 20, 21].

It is well known that certain proteins, including myoglobin, called serum cardiac markers, are released into the bloodstream in large quantities from necrotic cardiac muscle cells after myocardial infarction [22–24]. As direct samples from the ischaemic region are not readily obtainable, *in situ* studies of this protein in the cardiac ischaemic region have been limited [25]. We found myoglobin to be down-regulated in left ventricular tissue of patients with ICM. These results are consistent with those published by Dohko *et al*. in an experimental model of HF [12]. Myoglobin is not only important in intracellular oxygen supply but also constitutes a key element that influences various redox pathways in the muscle cell [26]. It seems likely that the multifunctional character of this protein creates an environment characterized by a tightly adapted aerobic mitochondrial respiration and low levels of free radicals, and thus plays an essential and beneficial role within the myocardium [27]. The underlying mechanisms by which myoglobin content decreases in failing myocardium are unknown but the decrease in this protein may contribute to the imbalance between energy production and energy expenditure in HF.

Myocytes compensate for the alteration in mitochondrial function by an increase in glycolysis-related proteins [28]. Changes in the expression of a variety of proteins involved in energy metabolism have been detected in HF by performing proteome analysis [29] and our results are in concordance with these studies. We found alterations in different proteins associated with metabolism in patients with ICM and DCM. Moreover, we have shown that some of them are overexpressed in both aetiologies, such as G3P and dihydrolipoyl dehydrogenase. The G3P is an ubiqitously expressed glycolytic enzyme essential for efficient energy production [30] and it is involved in cellular hypoxic and oxidative response [31], apoptosis [32], microtubule organization [33] and RNA-binding [34]. Increased levels of this protein have been found in spontaneous canine DCM [35] and in other experimental models of HF in which myocardial infarction was induced [12]. However, for the first time to the best of our knowledge, we have identified high levels of G3P in left ventricular tissue from patients with HF and we decided to focus on it. Furthermore, these results question the uselfulness of this protein as loading control in patients with HF. Immunofluorescence studies showed that G3P intensity was higher in the nucleus than in the cytoplasm in the ischaemic and dilated hearts. Apoptosis plays an important role in the remodelling of the myocardium in response to haemodynamic load [36] and HF is associated with increased free radical production, which leads to a state of oxidative stress [37]. There is evidence indicating that nuclear translocation of G3P plays a role in apoptosis and oxidative stress in neurodegenerative diseases, probably related to the activity of this protein as a DNA repair enzyme or as a nuclear carrier for pro-apoptotic molecules [38–41]. In this regard, we can think that in HF patients this translocation of G3P from the cytosol to the nucleus could also occur.

HSP71 is a protein involved in cellular recovery, survival and maintenance of cellular function [42]. Reports on the levels of HSP71 in HF patients due to ICM and DCM are contradictory. Specifically, it was reported that HSP71 levels were not increased significantly in DCM failing hearts compared with non-failing hearts [43]. Aversely, in a posterior study high levels of HSP71 were documented in the myocardium of HF patients with DCM [44]. In addition, increased serum levels of HSP71 were associated with a low prevalence of ICM [45], while other authors have shown increased serum HSP71 levels associated with a higher prevalence of ICM and DCM [46]. Corbett *et al*. showed increased expression of this protein in ICM [7]. Wei *et al*. demonstrated significant up-regulation of cardiac and serum HSP71 in ICM and DCM [8] and they observed in cultured cardiomyocytes pretreated with hypoxia to mimic the *in vivo* conditions of the myocardium in response to myocardial damage, a significantly elevation of intracellular and extracellular HSP71. Our results indicate that HSP71 is increased only in the ischaemic group. In addition, immunofluorescence studies showed that the ischaemic group had increased nuclear intensity. The expression of HSPs is induced by heat and by a variety of other cellular stresses including ischaemia and hypoxia. The heat shock or stress response is characterized by both an increase in transcription and translation of HSP70 family proteins, and nuclear translocation of the protein [47–49]. Studies have shown that cardiac ischaemia causes transient nuclear accumulation of HSP71 and HSP72 [47, 48]. These works indicate that HSPs may stabilize damaged proteins found within the nucleus after an acute stress, such as ischaemia. With the aim of screening differences between the two pathological groups, we compared them, noting that most of the altered proteins that were common in previous comparatives of each aetiology (pathological group *versus* controls) do appear in these comparative, no statistical difference was found (e.g. CRYAB, G3P). Moreover, we detected that proteins differentially expressed in CNT *versus* ICM or CNT *versus* DCM, were also altered in ICM *versus* DCM and in the same way. However, the most remarkable finding of the comparison of both aetiologies was the differential expression of proteins that did not appear altered when compared with the CNT group, such as proteins involved in cell structure and muscle contraction, including actin alpha cardiac muscle, down-regulated in patients with DCM. Alterations in the expression of these proteins affect myocardial contractility, and it has been found that actin dysfunction could lead to DCM [50].

It is noteworthy that in the present study most of the protein identified common in both aetiologies are interconnected on the same network that primarily involves structural and metabolic proteins and enzymes or transporters involved in respiratory chain. Interestingly, most of those proteins are related with cell death, specifically G3P increases apoptosis of eukariotic cells [51] and CRYAB is involved in inhibition of apoptosis and decreases necrosis of ischaemic cardiomyocites in cell culture [52, 53]. These proteins are related directly with huntingtin and this calcium channel regulator plays an important role in cell death and apoptosis in the brain. This interaction reaffirmed the implication of G3P and CRYAB in the apoptosis process in other cell types. Myocardial apoptosis represents a potential pathophysiological mechanism in HF progression [54], in this sense we can think that our results are consistent with previous data as proteins such as G3P increase and CRYAB decrease in our pathological samples.

One limitation of this study is the intrinsic variability of the samples, given they originate from human hearts, whose conditions (treatment they undergo) are not as standardized as those of studies using cell cultures. However, in our study most patients in both groups (ischaemic and dilated) received drugs like diuretics, aldosterone-antagonists, β-blockers, angiotensin-converting enzyme inhibitors or digoxin. Thus, it is very unlikely that the pharmacological treatment would contribute to the different protein profiles observed. Moreover, no statistically significant changes were found in the different drugs administered according to aetiology of HF, except for statins. Nevertheless, patients treated with statins had a similar protein profile compared with those not treated with this drug (data not shown).

In addition, there are limitations inherent to 2DE, such as the under-representation of very hydrophobic proteins, principally membrane proteins, and those above 100 kD. The use of zoom gels for the basic region of the proteome (pH 6–11) could expand the proteome study, allowing the identification of novel proteins. This is why we failed to identify cytokine proteins (IL-6, TNF-alpha, etc.), receptor expression (sTNF-R1, sTNF-R2, sFAS, etc.) and well-established HF markers, for example, BNP and NT-proBNP, which we determined in a previous study performed with Western blot techniques and mRNA expression quantified by quantitative real-time polymerase chain reaction (RT-PCR) in the same cardiac tissue [55]. Apart from this, we have identified a great number of mitochondrial proteins and it could be interesting to isolate mitochondria from left ventricular tissue and submit them to 2DE to characterize proteins related with this organelle.

In the present study, we have shown by proteomics analysis that HF is associated with changes in proteins involved in cellular stress response, respiratory chain and cardiac metabolism. Although we have found altered expression of eleven proteins common to both ischaemic and dilated aetiology, we also observed different proteins altered in both groups. Furthermore, we have shown that seven of these eleven proteins are involved in cell death and apoptosis processes, and therefore in HF progression. Understanding the proteome will offer the real possibility of knowing the function of the cardiovascular system in all of its complexity that should result in the discovery of new diagnostic or prognostic biomarkers and the identification of potential drug targets for the development of new therapeutic approaches for combating HF.

## Supporting information

**Table S1** Additional data on MS protein identification of ICM spots with differential expression by MALDI-MS

**Table S2** Additional data on MS protein identification of DCM spots with differential expression by MALDI-MS

**Table S3** Additional data on MS protein identification of ICM and DCM spots altered in the comparison of both pathologies by MALDI-MS
