# Supplementary material for: Cardiac protein changes in ischaemic and dilated cardiomyopathy: a proteomic study of human left ventricular tissue
Source: J Cell Mol Med. 2012 Sep 26;16(10):2471–86. doi: 10.1111/j.1582-4934.2012.01565.x (PMC3823441; doi:10.1111/j.1582-4934.2012.01565.x)
Supplement: Supplementary file 1 [file jcmm0016-2471-SD1.doc]

**Supplementary table 1:** Additional data on MS protein identification of ICM spots with differential expression by MALDI-MS. Peptides submitted to MS/MS, as an additional analysis that led to the same protein identification, are marked with an asterisk.

| **Spot** | **N^a^ / %^b^** | **Mascot Score** | **Peptides identified by MS** | | **Identified protein** | | **Accession number** | **Expected Mw** | **Expected pI** |
| --- | --- | --- | --- | --- | --- | --- | --- | --- | --- |
|  |  |  | **M+H** | **Sequence** |  |  |  |  |  |
| 29 | 8 / 43 | 248 | 857.41 | YVDMSVK | ATP synthase subunit O, mitochonrial | | ATPO_HUMAN | 20875.49 | 9.81 |
|  |  |  | 861.47 | SLNDITAK |  |  |  |  |  |
|  |  |  | 1106.62 | *SFLSQGQVLK |  |  |  |  |  |
|  |  |  | 1145.58 | *YATALYSAASK |  | |  |  |  |
|  |  |  | 1160.67 | *VAASVLNPYVK |  |  |  |  |  |
|  |  |  | 1274.68 | TDPSILGGMIVR |  |  |  |  |  |
|  |  |  | 1582.91 | LVRPPVQVYGIEGR |  | |  |  |  |
|  |  |  | 2083.99 | LSNTQGVVSAFSTMMSVHR |  | |  |  |  |
| 43 | 9 / 40 | 342 | 702.43 | *IFVPAR | NADH dehydrogenase [ubiquinone] iron-sulfur protein 4, mitochondrial | | NDUS4_HUMAN | 15385.33 | 9.36 |
|  |  |  | 814.34 | MEFDTR |  |  |  |  |  |
|  |  |  | 995.47 | EDAVSFAEK |  |  |  |  |  |
|  |  |  | 1128.51 | WKMEFDTR |  |  |  |  |  |
|  |  |  | 1173.53 | *SYGANFSWNK |  |  |  |  |  |
|  |  |  | 1222.55 | NNMQSGVNNTK |  |  |  |  |  |
|  |  |  | 1268.55 | *NGWSYDIEER |  |  |  |  |  |
|  |  |  | 1329.63 | SYGANFSWNKR |  |  |  |  |  |
|  |  |  | 1665.91 | *LDITTLTGVPEEHIK |  | |  |  |  |
| 66 | 9 / 42 | 317 | 838.50 | ALPAPIEK | Ig gamma-1 chain C region | | IGHG1_HUMAN | 36105.91 | 8.46 |
|  |  |  | 851.43 | DTLMISR |  |  |  |  |  |
|  |  |  | 1286.67 | *EPQVYTLPPSR |  |  |  |  |  |
|  |  |  | 1677.80 | *FNWYVDGVEVHNAK |  | |  |  |  |
|  |  |  | 1808.01 | *VVSVLTVLHQDWLNGK |  | |  |  |  |
|  |  |  | 1873.92 | *TTPPVLDSDGSFFLYSK |  | |  |  |  |
|  |  |  | 2139.03 | *TPEVTCVVVDVSHEDPEVK |  | |  |  |  |
|  |  |  | 2544.13 | GFYPSDIAVEWESNGQPENNYK |  | |  |  |  |
|  |  |  | 2844.46 | THTCPPCPAPELLGGPSVFLFPPKPK |  | |  |  |  |
| 86 | 8 / 36 | 295 | 805.43 | VGVNGFGR | Glyceraldehyde-3-phosphate dehydrogenase | | G3P_HUMAN | 35922.02 | 8.58 |
|  |  |  | 811.41 | LTGMAFR |  |  |  |  |  |
|  |  |  | 1530.79 | *VPTANVSVVDLTCR |  |  |  |  |  |
|  |  |  | 1613.90 | *LVINGNPITIFQER |  |  |  |  |  |
|  |  |  | 1763.80 | *LISWYDNEFGYSNR |  |  |  |  |  |
|  |  |  | 2245.10 | VIISAPSADAPMFVMGVNHEK |  |  |  |  |  |
|  |  |  | 2293.03 | WGDAGAEYVVESTGVFTTMEK |  | |  |  |  |
|  |  |  | 2611.35 | VIHDNFGIVEGLMTTVHAITATQK |  | |  |  |  |
| 99 | 17 / 64 | 598 | 739.36 | YDNSLK | Glyceraldehyde-3-phosphate dehydrogenase | | G3P_HUMAN | 35922.02 | 8.58 |
|  |  |  | 760.38 | TVDGPSGK |  |  |  |  |  |
|  |  |  | 805.43 | VGVNGFGR |  |  |  |  |  |
|  |  |  | 811.41 | LTGMAFR |  |  |  |  |  |
|  |  |  | 829.44 | QASEGPLK |  | |  |  |  |
|  |  |  | 909.49 | AGAHLQGGAK |  | |  |  |  |
|  |  |  | 1411.79 | GALQNIIPASTGAAK |  | |  |  |  |
|  |  |  | 1530.79 | *VPTANVSVVDLTCR |  | |  |  |  |
|  |  |  | 1613.90 | *LVINGNPITIFQER |  | |  |  |  |
|  |  |  | 1763.80 | *LISWYDNEFGYSNR |  | |  |  |  |
|  |  |  | 1833.92 | IISNASCTTNCLAPLAK |  | |  |  |  |
|  |  |  | 2229.10 | VIISAPSADAPMFVMGVNHEK |  | |  |  |  |
|  |  |  | 2245.10 | VIISAPSADAPMFVMGVNHEK |  | |  |  |  |
|  |  |  | 2293.03 | WGDAGAEYVVESTGVFTTMEK |  | |  |  |  |
|  |  |  | 2401.20 | RVIISAPSADAPMFVMGVNHEK |  | |  |  |  |
|  |  |  | 2611.35 | *VIHDNFGIVEGLMTTVHAITATQK |  | |  |  |  |
|  |  |  | 3340.55 | VDIVAINDPFIDLNYMVYMFQYDSTHGK |  | |  |  |  |
| 102 | 8 / 42 | 308 | 1135.59 | *NGFLLDGFPR | Adenylate kinase 2, mitochondrial | | KAD2_HUMAN | 26346.55 | 7.85 |
|  |  |  | 1355.68 | APSVPAAEPEYPK |  |  |  |  |  |
|  |  |  | 1519.79 | LVSDEMVVELIEK |  |  |  |  |  |
|  |  |  | 1629.82 | EPMKDDITGEPLIR |  |  |  |  |  |
|  |  |  | 1817.01 | *LDSVIEFSIPDSLLIR |  |  |  |  |  |
|  |  |  | 1997.01 | LQAYHTQTTPLIEYYR |  |  |  |  |  |
|  |  |  | 2022.92 | LAENFCVCHLATGDMLR |  |  |  |  |  |
|  |  |  | 2074.14 | *EKLDSVIEFSIPDSLLIR |  | |  |  |  |
| 115 | 6 / 30 | 72 | 1135.59 | *NGFLLDGFPR | Adenylate kinase 2, mitochondrial | | KAD2_HUMAN | 26346.55 | 7.85 |
|  |  |  | 1193.62 | AMVASGSELGKK |  |  |  |  |  |
|  |  |  | 1817.01 | LDSVIEFSIPDSLLIR |  | |  |  |  |
|  |  |  | 1997.01 | LQAYHTQTTPLIEYYR |  | |  |  |  |
|  |  |  | 2022.92 | LAENFCVCHLATGDMLR |  | |  |  |  |
|  |  |  | 2074.14 | EKLDSVIEFSIPDSLLIR |  | |  |  |  |
| 141 | 7 / 33 | 200 | 718.39 | EQWKK | NADH dehydrogenase [ubiquinone] 1 beta subcomplex 9 | | NDUB9_HUMAN | 21699.72 | 8.59 |
|  |  |  | 746.39 | REQWK |  |  |  |  |  |
|  |  |  | 976.44 | YFACLMR |  |  |  |  |  |
|  |  |  | 1121.50 | AMYPDYFAK |  |  |  |  |  |
|  |  |  | 1214.57 | HLESWCVQR |  | |  |  |  |
|  |  |  | 1258.54 | *EAEEEFWYR |  | |  |  |  |
|  |  |  | 2101.09 | *QLQEETPPGGPLTEALPPAR |  | |  |  |  |
| 146 | 8 / 37 | 330 | 922.42 | *YDNFAHR | Protein NipSnap homolog 3A | | NPS3A_HUMAN | 28466.61 | 9.21 |
|  |  |  | 1191.60 | MNTVFHIWK |  |  |  |  |  |
|  |  |  | 1309.70 | AVHAHVNLGYTK |  |  |  |  |  |
|  |  |  | 1316.58 | MNEFLENFEK |  | |  |  |  |
|  |  |  | 1337.62 | *QYDGIFYEFR |  | |  |  |  |
|  |  |  | 1575.83 | *LVGVFHTEYGALNR |  | |  |  |  |
|  |  |  | 1598.77 | *VHVLWWNESADSR |  | |  |  |  |
|  |  |  | 1894.91 | *TAHSELVGYWSVEFGGR |  | |  |  |  |
| 196 | 21 / 41 | 463 | 757.41 | NWKPGR | Electron transfer flavoprotein-ubiquinone oxidoreductase, mitochondrial | | ETFD_HUMAN | 64675.68 | 6.52 |
|  |  |  | 766.41 | ELYSVR |  |  |  |  |  |
|  |  |  | 819.41 | NSWVWK |  |  |  |  |  |
|  |  |  | 934.47 | ELFPDWK |  |  |  |  |  |
|  |  |  | 1031.54 | ELWVIDEK |  |  |  |  |  |
|  |  |  | 1036.45 | WEGVNMER |  | |  |  |  |
|  |  |  | 1260.66 | ALNEGGFQSIPK |  | |  |  |  |
|  |  |  | 1264.67 | *ITTHYTIYPR |  | |  |  |  |
|  |  |  | 1291.63 | *NLSIYDGPEQR |  | |  |  |  |
|  |  |  | 1294.65 | VDHTVGWPLDR |  | |  |  |  |
|  |  |  | 1428.77 | HHPSIRPTLEGGK |  | |  |  |  |
|  |  |  | 1450.70 | ANCEPQTYGIGLK |  | |  |  |  |
|  |  |  | 1631.83 | TIGLHVTEYEDNLK |  | |  |  |  |
|  |  |  | 1742.94 | WKHHPSIRPTLEGGK |  | |  |  |  |
|  |  |  | 1984.99 | AAQIGAHTLSGACLDPGAFK |  | |  |  |  |
|  |  |  | 2099.11 | *FAEEADVVIVGAGPAGLSAAVR |  | |  |  |  |
|  |  |  | 2121.09 | LTFPGGLLIGCSPGFMNVPK |  | |  |  |  |
|  |  |  | 2174.00 | *FCPAGVYEFVPVEQGDGFR |  | |  |  |  |
|  |  |  | 2188.06 | DDSIPVNRNLSIYDGPEQR |  | |  |  |  |
|  |  |  | 2230.22 | IPVPILPGLPMNNHGNYIVR |  | |  |  |  |
|  |  |  | 2250.16 | SGILAAESIFNQLTSENLQSK |  | |  |  |  |
|  | 9 / 27 | 202 | 734.42 | *AAQLGFK | Dihydrolipoyl dehydrogenase, mitochondrial | | DLDH_HUMAN | 50174.62 | 6.50 |
|  |  |  | 909.46 | *FPFAANSR |  |  |  |  |  |
|  |  |  | 1524.74 | *VCHAHPTLSEAFR |  |  |  |  |  |
|  |  |  | 1567.83 | NLGLEELGIELDPR |  |  |  |  |  |
|  |  |  | 1581.76 | SEEQLKEEGIEYK |  | |  |  |  |
|  |  |  | 1717.90 | AEVITCDVLLVCIGR |  | |  |  |  |
|  |  |  | 1986.08 | MVVIGAGVIGVELGSVWQR |  | |  |  |  |
|  |  |  | 1995.07 | IPNIYAIGDVVAGPMLAHK |  | |  |  |  |
|  |  |  | 3370.64 | VLGAHILGPGAGEMVNEAALALEYGASCEDIAR |  | |  |  |  |
| 197 | 14 / 31 | 238 | 707.38 | QAVAYR | ATP synthase subunit alpha, mitochondrial | | ATPA_HUMAN | 55209.32 | 8.28 |
|  |  |  | 716.46 | LTELLK |  |  |  |  |  |
|  |  |  | 723.45 | APGIIPR |  |  |  |  |  |
|  |  |  | 815.47 | GPIGSKTR |  | |  |  |  |
|  |  |  | 892.49 | LELAQYR |  | |  |  |  |
|  |  |  | 1026.59 | AVDSLVPIGR |  | |  |  |  |
|  |  |  | 1553.74 | *EAYPGDVFYLHSR |  | |  |  |  |
|  |  |  | 1564.86 | QAVAYRQMSLLLR |  | |  |  |  |
|  |  |  | 1575.79 | ILGADTSVDLEETGR |  | |  |  |  |
|  |  |  | 1624.89 | *TGAIVDVPVGEELLGR |  | |  |  |  |
|  |  |  | 1683.79 | NVQAEEMVEFSSGLK |  | |  |  |  |
|  |  |  | 2325.15 | QGQYSPMAIEEQVAVIYAGVR |  | |  |  |  |
|  |  |  | 2338.17 | EVAAFAQFGSDLDAATQQLLSR |  | |  |  |  |
|  |  |  | 2367.26 | FENAFLSHVVSQHQALLGTIR |  | |  |  |  |
| 211 | 19 / 54 | 531 | 704.43 | VTGATKK | Dihydrolipoyl dehydrogenase, mitochondrial | | DLDH_HUMAN | 50174.62 | 6.50 |
|  |  |  | 734.42 | AAQLGFK |  |  |  |  |  |
|  |  |  | 749.39 | TVCIEK |  |  |  |  |  |
|  |  |  | 909.46 | *FPFAANSR |  |  |  |  |  |
|  |  |  | 912.54 | GRIPVNTR |  | |  |  |  |
|  |  |  | 936.45 | GIEMSEVR |  | |  |  |  |
|  |  |  | 972.53 | VVHVNGYGK |  | |  |  |  |
|  |  |  | 1007.52 | EANLAASFGK |  | |  |  |  |
|  |  |  | 1127.66 | ALTGGIAHLFK |  | |  |  |  |
|  |  |  | 1524.74 | *VCHAHPTLSEAFR |  | |  |  |  |
|  |  |  | 1567.83 | *NLGLEELGIELDPR |  | |  |  |  |
|  |  |  | 1581.76 | *SEEQLKEEGIEYK |  | |  |  |  |
|  |  |  | 1717.90 | *AEVITCDVLLVCIGR |  | |  |  |  |
|  |  |  | 1819.87 | NETLGGTCLNVGCIPSK |  | |  |  |  |
|  |  |  | 1986.08 | MVVIGAGVIGVELGSVWQR |  | |  |  |  |
|  |  |  | 1995.07 | IPNIYAIGDVVAGPMLAHK |  | |  |  |  |
|  |  |  | 2530.29 | LGADVTAVEFLGHVGGVGIDMEISK |  | |  |  |  |
|  |  |  | 3359.78 | NILIATGSEVTPFPGITIDEDTIVSSTGALSLK |  | |  |  |  |
|  |  |  | 3370.64 | VLGAHILGPGAGEMVNEAALALEYGASCEDIAR |  | |  |  |  |
| 228 | 18 / 50 | 759 | 776.44 | *AGNVIFR | Succinyl-CoA :3-ketoacid-coenzyme A transferase 1, mitochondrial | | SCOT1_HUMAN | 52089.89 | 6.00 |
|  |  |  | 793.41 | AVFDVDK |  |  |  |  |  |
|  |  |  | 944.48 | SAKPGDDVR |  |  |  |  |  |
|  |  |  | 1039.47 | NFNLPMCK |  |  |  |  |  |
|  |  |  | 1077.51 | LMPMQQIAN |  |  |  |  |  |
|  |  |  | 1100.61 | DGSVAIASKPR |  | |  |  |  |
|  |  |  | 1168.59 | FYTDPVEAVK |  | |  |  |  |
|  |  |  | 1380.66 | YGDLANWMIPGK |  | |  |  |  |
|  |  |  | 1581.78 | MVKGMGGAMDLVSSAK |  | |  |  |  |
|  |  |  | 1617.72 | MVSSYVGENAEFER |  | |  |  |  |
|  |  |  | 1633.72 | *MVSSYVGENAEFER |  | |  |  |  |
|  |  |  | 1675.81 | GGHVDLTMLGAMQVSK |  | |  |  |  |
|  |  |  | 2101.14 | *GLTAVSNNAGVDNFGLGLLLR |  | |  |  |  |
|  |  |  | 2233.13 | *QYLSGELEVELTPQGTLAER |  | |  |  |  |
|  |  |  | 2378.20 | *EFNGQHFILEEAITGDFALVK |  | |  |  |  |
|  |  |  | 2421.16 | ETVTILPGASFFSSDESFAMIR |  | |  |  |  |
|  |  |  | 2595.31 | *AGGAGVPAFYTPTGYGTLVQEGGSPIK |  | |  |  |  |
|  |  |  | 3560.83 | AAETTVVEVEEIVDIGAFAPEDIHIPQIYVHR |  | |  |  |  |
| 239 | 10 / 41 | 259 | 793.36 | REGMER | Malate dehydrogenase, cytoplasmic | | MDHC_HUMAN | 36294.93 | 6.89 |
|  |  |  | 1026.47 | ENFSCLTR |  |  |  |  |  |
|  |  |  | 1164.60 | *GEFVTTVQQR |  |  |  |  |  |
|  |  |  | 1393.71 | *FVEGLPINDFSR |  |  |  |  |  |
|  |  |  | 1401.74 | *DLDVAILVGSMPR |  |  |  |  |  |
|  |  |  | 1450.74 | DVIATDKEDVAFK |  |  |  |  |  |
|  |  |  | 1751.89 | EVGVYEALKDDSWLK |  | |  |  |  |
|  |  |  | 1757.92 | VIVVGNPANTNCLTASK |  | |  |  |  |
|  |  |  | 2280.12 | NVIIWGNHSSTQYPDVNHAK |  | |  |  |  |
|  |  |  | 2585.40 | VLVTGAAGQIAYSLLYSIGNGSVFGK |  | |  |  |  |
| 318 | 8 / 51 | 169 | 889.47 | NVLTESAR | ES1 protein homolog, mitochondrial | | ES1_HUMAN | 24016.57 | 6.63 |
|  |  |  | 946.42 | GQPSEGESR |  |  |  |  |  |
|  |  |  | 1153.58 | EVVEAHVDQK |  |  |  |  |  |
|  |  |  | 1206.62 | WPYAGTAEAIK |  | |  |  |  |
|  |  |  | 2405.19 | GGAEVQIFAPDVPQMHVIDHTK |  |  |  |  |  |
|  |  |  | 2442.24 | *ITDLANLSAANHDAAIFPGGFGAAK |  | |  |  |  |
|  |  |  | 2821.35 | VVTTPAFMCETALHYIHDGIGAMVR |  |  |  |  |  |
|  |  |  | 2966.54 | VALVLSGCGVYDGTEIHEASAILVHLSR |  | |  |  |  |
| 326 | 15 / 85 | 563 | 854.51 | *DLAPIGIR | 3-hydroxyacyl-CoA dehydrogenase type-2 | | HCD2_HUMAN | 26791.89 | 7.89 |
|  |  |  | 1200.68 | GGIVGMTLPIAR |  |  |  |  |  |
|  |  |  | 1331.63 | *GQTHTLEDFQR |  |  |  |  |  |
|  |  |  | 1459.73 | KGQTHTLEDFQR |  |  |  |  |  |
|  |  |  | 1473.77 | VDVAVNCAGIAVASK |  |  |  |  |  |
|  |  |  | 1606.86 | *VLDVNLMGTFNVIR |  | |  |  |  |
|  |  |  | 1621.82 | *VCNFLASQVPFPSR |  | |  |  |  |
|  |  |  | 1721.82 | LGNNCVFAPADVTSEK |  | |  |  |  |
|  |  |  | 1801.81 | *LVAGEMGQNEPDQGGQR |  | |  |  |  |
|  |  |  | 1813.02 | *GLVAVITGGASGLGLATAER |  | |  |  |  |
|  |  |  | 1849.91 | KLGNNCVFAPADVTSEK |  | |  |  |  |
|  |  |  | 2101.16 | VMTIAPGLFGTPLLTSLPEK |  | |  |  |  |
|  |  |  | 2195.17 | LVGQGASAVLLDLPNSGGEAQAK |  | |  |  |  |
|  |  |  | 2439.25 | GVIINTASVAAFEGQVGQAAYSASK |  | |  |  |  |
|  |  |  | 2878.51 | LGDPAEYAHLVQAIIENPFLNGEVIR |  | |  |  |  |
| 333 | 16 / 74 | 423 | 700.44 | *TIPITR | Alpha-crystallin B chain | | CRYAB_HUMAN | 20158.91 | 6.76 |
|  |  |  | 716.38 | EFHRK |  |  |  |  |  |
|  |  |  | 772.39 | QVSGPER |  |  |  |  |  |
|  |  |  | 900.49 | KQVSGPER |  |  |  |  |  |
|  |  |  | 921.50 | FSVNLDVK |  | |  |  |  |
|  |  |  | 986.49 | HFSPEELK |  | |  |  |  |
|  |  |  | 1088.51 | QDEHGFISR |  | |  |  |  |
|  |  |  | 1140.63 | EEKPAVTAAPK |  | |  |  |  |
|  |  |  | 1165.66 | VLGDVIEVHGK |  | |  |  |  |
|  |  |  | 1192.63 | DRFSVNLDVK |  | |  |  |  |
|  |  |  | 1374.71 | *RPFFPFHSPSR |  | |  |  |  |
|  |  |  | 1388.73 | MDIAIHHPWIR |  | |  |  |  |
|  |  |  | 1404.72 | MDIAIHHPWIR |  | |  |  |  |
|  |  |  | 1496.68 | APSWFDTGLSEMR |  | |  |  |  |
|  |  |  | 2624.38 | *IPADVDPLTITSSLSSDGVLTVNGPR |  | |  |  |  |
|  |  |  | 2943.54 | YRIPADVDPLTITSSLSSDGVLTVNGPR |  | |  |  |  |
| 344 | 6 / 48 | 242 | 748.44 | *ALELFR | Myoglobin | | MYG_HUMAN | 17052.61 | 7.29 |
|  |  |  | 910.46 | GHPETLEK |  |  |  |  |  |
|  |  |  | 1350.81 | HGATVLTALGGILK |  |  |  |  |  |
|  |  |  | 1531.66 | *HPGDFGADAQGAMNK |  |  |  |  |  |
|  |  |  | 1632.87 | *VEADIPGHGQEVLIR |  |  |  |  |  |
|  |  |  | 1970.03 | YLEFISECIIQVLQSK |  | |  |  |  |
|  | 9 / 52 | 156 | 705.38 | ALSTGEK | Peptidyl-prolyp cis-trans isomerase A | | PPIA_HUMAN | 17881.30 | 7.82 |
|  |  |  | 737.36 | *TAENFR |  |  |  |  |  |
|  |  |  | 1055.54 | VSFELFADK |  |  |  |  |  |
|  |  |  | 1154.57 | FEDENFILK |  | |  |  |  |
|  |  |  | 1247.63 | KITIADCGQLE |  | |  |  |  |
|  |  |  | 1310.57 | EGMNIVEAMER |  | |  |  |  |
|  |  |  | 1537.73 | VKEGMNIVEAMER |  | |  |  |  |
|  |  |  | 1614.74 | IIPGFMCQGGDFTR |  | |  |  |  |
|  |  |  | 1946.00 | *VNPTVFFDIAVDGEPLGR |  | |  |  |  |
| 352 | 7 / 88 | 272 | 713.42 | VPAGLEK | D-dopachrome decarboxylase | | DOPD_HUMAN | 12580.57 | 7.25 |
|  |  |  | 901.47 | ELALGQDR |  |  |  |  |  |
|  |  |  | 1351.70 | FFPLESWQIGK |  |  |  |  |  |
|  |  |  | 1450.71 | SHSAHFFEFLTK |  | |  |  |  |
|  |  |  | 1499.79 | PFLELDTNLPANR |  | |  |  |  |
|  |  |  | 1513.82 | LCAAAASILGKPADR |  | |  |  |  |
|  |  |  | 3872.95 | VNVTVRPGLAMALSGSTEPCAQLSISSIGVVGTAEDNR |  | |  |  |  |
| 363 | 23 / 43 | 293 | 788.43 | VSAQEVR | Ezrin | | EZRI_HUMAN | 69281.61 | 5.95 |
|  |  |  | 823.51 | PKPINVR |  | |  |  |  |
|  |  |  | 848.47 | GFPTWLK |  | |  |  |  |
|  |  |  | 914.53 | IALLEEAR |  | |  |  |  |
|  |  |  | 976.55 | QLFDQVVK |  | |  |  |  |
|  |  |  | 987.51 | ALQLEEER |  | |  |  |  |
|  |  |  | 1002.52 | ELSEQIQR |  | |  |  |  |
|  |  |  | 1063.55 | EKEELMLR |  | |  |  |  |
|  |  |  | 1104.58 | *IGFPWSEIR |  | |  |  |  |
|  |  |  | 1120.56 | QRIDEFEAL |  | |  |  |  |
|  |  |  | 1175.61 | IQVWHAEHR |  | |  |  |  |
|  |  |  | 1182.59 | *APDFVFYAPR |  | |  |  |  |
|  |  |  | 1310.69 | *KAPDFVFYAPR |  | |  |  |  |
|  |  |  | 1445.80 | QLLTLSSELSQAR |  | |  |  |  |
|  |  |  | 1488.78 | RKPDTIEVQQMK |  | |  |  |  |
|  |  |  | 1493.69 | THNDIIHNENMR |  | |  |  |  |
|  |  |  | 1651.82 | SQEQLAAELAEYTAK |  | |  |  |  |
|  |  |  | 1669.80 | EVWYFGLHYVDNK |  | |  |  |  |
|  |  |  | 1809.84 | ILQLCMGNHELYMR |  | |  |  |  |
|  |  |  | 1962.95 | IAQDLEMYGINYFEIK |  | |  |  |  |
|  |  |  | 2038.00 | FYPEDVAEELIQDITQK |  | |  |  |  |
|  |  |  | 2082.01 | VTTMDAELEFAIQPNTTGK |  | |  |  |  |
|  |  |  | 2823.41 | EGILSDEIYCPPETAVLLGSYAVQAK |  | |  |  |  |
| 460 | 3 / 50 | 122 | 1797.90 | *SGTASVVCLLNNFYPR | Ig kappa chain C region | | IGKC_HUMAN | 11608.86 | 5.58 |
|  |  |  | 1875.93 | *VYACEVTHQGLSSPVTK |  | |  |  |  |
|  |  |  | 2135.97 | VDNALQSGNSQESVTEQDSK |  | |  |  |  |
| 476 | 6 / 20 | 141 | 730.42 | AAAAASLR | Cytochrome c1, heme protein, mitochondrial | | CY1_HUMAN | 27352.36 | 6.49 |
|  |  |  | 732.41 | SGLSRGR |  |  |  |  |  |
|  |  |  | 844.50 | LAYRPPK |  |  |  |  |  |
|  |  |  | 1298.71 | GLLSSLDHTSIR |  | |  |  |  |
|  |  |  | 1670.89 | *AANNGALPPDLSYIVR |  | |  |  |  |
|  |  |  | 1914.94 | *LFDYFPKPYPNSEAAR |  | |  |  |  |
| 489 | 9 / 31 | 91 | 816.51 | *GKPLFVR | Cytochrome b-c1 complex subunit Rieske, mitochondrial | | UCRI_HUMAN | 49128.60 | 5.43 |
|  |  |  | 995.45 | DPQHDLDR |  |  |  |  |  |
|  |  |  | 1012.47 | *VPDFSEYR |  |  |  |  |  |
|  |  |  | 1060.60 | RLEVLDSTK |  |  |  |  |  |
|  |  |  | 1168.57 | VPDFSEYRR |  | |  |  |  |
|  |  |  | 1614.83 | *EIEQEAAVELSQLR |  | |  |  |  |
|  |  |  | 1904.02 | GFSYLVTGVTTVGVAYAAK |  | |  |  |  |
|  |  |  | 2032.11 | *KGFSYLVTGVTTVGVAYAAK |  | |  |  |  |
|  |  |  | 2126.08 | NAVTQFVSSMSASADVLALAK |  | |  |  |  |
| 490 | 4 / 13 | 103 | 763.41 | MLSVASR | Cytochrome b-c1 complex subunit Rieske, mitochondrial | | UCRI_HUMAN | 49128.60 | 5.43 |
|  |  |  | 816.51 | GKPLFVR |  |  |  |  |  |
|  |  |  | 1012.47 | *VPDFSEYR |  |  |  |  |  |
|  |  |  | 1614.83 | *EIEQEAAVELSQLR |  | |  |  |  |
| 534 | 23 / 50 | 715 | 705.39 | STGKANK | Heat shock 70 kDa protein 1 | | HSP71_HUMAN | 69921.04 | 5.48 |
|  |  |  | 801.42 | DNNLLGR |  |  |  |  |  |
|  |  |  | 1109.57 | *LLQDFFNGR |  |  |  |  |  |
|  |  |  | 1137.55 | YKAEDEVQR |  |  |  |  |  |
|  |  |  | 1197.69 | *DAGVIAGLNVLR |  |  |  |  |  |
|  |  |  | 1228.63 | VEIIANDQGNR |  |  |  |  |  |
|  |  |  | 1261.66 | LVNHFVEEFK |  |  |  |  |  |
|  |  |  | 1303.60 | NALESYAFNMK |  |  |  |  |  |
|  |  |  | 1315.60 | *FEELCSDLFR |  |  |  |  |  |
|  |  |  | 1417.76 | LVNHFVEEFKR |  | |  |  |  |
|  |  |  | 1465.81 | AQIHDLVLVGGSTR |  | |  |  |  |
|  |  |  | 1487.70 | *TTPSYVAFTDTER |  | |  |  |  |
|  |  |  | 1542.74 | ARFEELCSDLFR |  | |  |  |  |
|  |  |  | 1630.80 | AFYPEEISSMVLTK |  | |  |  |  |
|  |  |  | 1658.85 | NQVALNPQNTVFDAK |  | |  |  |  |
|  |  |  | 1675.73 | *ATAGDTHLGGEDFDNR |  | |  |  |  |
|  |  |  | 1680.85 | HWPFQVINDGDKPK |  | |  |  |  |
|  |  |  | 1687.90 | *IINEPTAAAIAYGLDR |  | |  |  |  |
|  |  |  | 2265.13 | AAAIGIDLGTTYSCVGVFQHGK |  | |  |  |  |
|  |  |  | 2786.36 | *QTQIFTTYSDNQPGVLIQVYEGER |  | |  |  |  |
|  |  |  | 2981.46 | TLSSSTQASLEIDSLFEGIDFYTSITR |  | |  |  |  |
|  |  |  | 3001.49 | *EIAEAYLGYPVTNAVITVPAYFNDSQR |  | |  |  |  |
|  |  |  | 3055.49 | ELEQVCNPIISGLYQGAGGPGPGGFGAQGPK |  | |  |  |  |
| 540 | 17 / 30 | 604 | 731.41 | LVGMPAK | Stress-70 protein, mitochondrial | | GRP75_HUMAN | 68759.00 | 5.44 |
|  |  |  | 863.40 | DNMALQR |  |  |  |  |  |
|  |  |  | 938.43 | YAEEDRR |  |  |  |  |  |
|  |  |  | 958.50 | VLENAEGAR |  | |  |  |  |
|  |  |  | 960.55 | DIKNVPFK |  | |  |  |  |
|  |  |  | 1148.55 | KDSETGENIR |  | |  |  |  |
|  |  |  | 1149.55 | RYDDPEVQK |  | |  |  |  |
|  |  |  | 1242.68 | *DAGQISGLNVLR |  | |  |  |  |
|  |  |  | 1290.68 | *VQQTVQDLFGR |  | |  |  |  |
|  |  |  | 1361.74 | *AQFEGIVTDLIR |  | |  |  |  |
|  |  |  | 1450.72 | TTPSVVAFTADGER |  | |  |  |  |
|  |  |  | 1462.76 | *SDIGEVILVGGMTR |  | |  |  |  |
|  |  |  | 1568.77 | QAVTNPNNTFYATK |  | |  |  |  |
|  |  |  | 1592.95 | *LLGQFTLIGIPPAPR |  | |  |  |  |
|  |  |  | 1694.85 | *NAVITVPAYFNDSQR |  | |  |  |  |
|  |  |  | 2055.96 | *STNGDTFLGGEDFDQALLR |  | |  |  |  |
|  |  |  | 2251.22 | VIAVYDLGGGTFDISILEIQK |  | |  |  |  |
| 628 | 16 / 66 | 349 | 700.29 | AFMCR | Glutathione  S-transferase Mu 3 | | GSTM3_HUMAN | 26428.40 | 5.37 |
|  |  |  | 736.39 | FEALEK |  |  |  |  |  |
|  |  |  | 737.44 | GLAHAIR |  |  |  |  |  |
|  |  |  | 971.46 | *FSWFAGEK |  | |  |  |  |
|  |  |  | 973.48 | QFSMFLGK |  | |  |  |  |
|  |  |  | 1015.59 | ITQSNAILR |  | |  |  |  |
|  |  |  | 1135.55 | CLDEFPNLK |  | |  |  |  |
|  |  |  | 1346.53 | YTCGEAPDYDR |  | |  |  |  |
|  |  |  | 1443.69 | IAAYLQSDQFCK |  | |  |  |  |
|  |  |  | 1494.73 | *VDIIENQVMDFR |  | |  |  |  |
|  |  |  | 1587.78 | LLLEFTDTSYEEK |  | |  |  |  |
|  |  |  | 1655.94 | *LKPQYLEELPGQLK |  | |  |  |  |
|  |  |  | 1743.88 | LLLEFTDTSYEEKR |  | |  |  |  |
|  |  |  | 1763.91 | IRVDIIENQVMDFR |  | |  |  |  |
|  |  |  | 1973.00 | *LTFVDFLTYDILDQNR |  | |  |  |  |
|  |  |  | 2008.08 | FKLDLDFPNLPYLLDGK |  | |  |  |  |
| 629 | 10 / 50 | 284 | 765.39 | AEDFRK | Peroxiredoxin-2 | | PRDX2_HUMAN | 21760.73 | 5.67 |
|  |  |  | 789.41 | SVDEALR |  |  |  |  |  |
|  |  |  | 810.44 | LSDYKGK |  |  |  |  |  |
|  |  |  | 924.44 | *TDEGIAYR |  | |  |  |  |
|  |  |  | 972.55 | IGKPAPDFK |  | |  |  |  |
|  |  |  | 1023.54 | LSEDYGVLK |  | |  |  |  |
|  |  |  | 1211.67 | *QITVNDLPVGR |  | |  |  |  |
|  |  |  | 1734.97 | *EGGLGPLNIPLLADVTR |  | |  |  |  |
|  |  |  | 1863.07 | *KEGGLGPLNIPLLADVTR |  | |  |  |  |
|  |  |  | 3243.66 | GKYVVLFFYPLDFTFVCPTEIIAFSNR |  | |  |  |  |
| 640 | 8 / 51 | 217 | 831.51 | VPFSLLR | Heat shock protein  beta-1 | | HSPB1_HUMAN | 22782.52 | 5.98 |
|  |  |  | 960.43 | DWYPHSR |  |  |  |  |  |
|  |  |  | 961.45 | GPSWDPFR |  | |  |  |  |
|  |  |  | 1104.51 | QDEHGYISR |  |  |  |  |  |
|  |  |  | 1163.62 | *LFDQAFGLPR |  | |  |  |  |
|  |  |  | 1783.92 | VSLDVNHFAPDELTVK |  |  |  |  |  |
|  |  |  | 1905.99 | *LATQSNEITIPVTFESR |  | |  |  |  |
|  |  |  | 3242.65 | KYTLPPGVDPTQVSSSLSPEGTLTVEAPMPK |  | |  |  |  |
| 649 | 13 / 64 | 610 | 720.40 | AVIFDR | Prohibin | | PHB_HUMAN | 29804.10 | 5.57 |
|  |  |  | 1058.52 | QVAQQEAER |  |  |  |  |  |
|  |  |  | 1062.51 | QVSDDLTER |  |  |  |  |  |
|  |  |  | 1149.59 | *FDAGELITQR |  | |  |  |  |
|  |  |  | 1185.66 | *DLQNVNITLR |  | |  |  |  |
|  |  |  | 1213.74 | VLPSITTEILK |  | |  |  |  |
|  |  |  | 1396.84 | *ILFRPVASQLPR |  | |  |  |  |
|  |  |  | 1444.66 | *IFTSIGEDYDER |  | |  |  |  |
|  |  |  | 1606.84 | *KLEAAEDIAYQLSR |  | |  |  |  |
|  |  |  | 1998.09 | *AAELIANSLATAGDGLIELR |  | |  |  |  |
|  |  |  | 2098.17 | *SRNITYLPAGQSVLLQLPQ |  | |  |  |  |
|  |  |  | 2119.14 | AATFGLILDDVSLTHLTFGK |  | |  |  |  |
|  |  |  | 2371.25 | *FGLALAVAGGVVNSALYNVDAGHR |  | |  |  |  |
| 678 | 8 / 80 | 266 | 744.39 | EFHRR | Heat shock protein beta-6 | | HSPB6_HUMAN | 17135.60 | 5.95 |
|  |  |  | 1156.60 | HFSPEEIAVK |  |  |  |  |  |
|  |  |  | 1231.65 | *VVGEHVEVHAR |  |  |  |  |  |
|  |  |  | 1578.74 | *HEERPDEHGFVAR |  | |  |  |  |
|  |  |  | 2557.40 | *APSVALPVAQVPTDPGHFSVLLDVK |  | |  |  |  |
|  |  |  | 2668.37 | FGEGLLEAELAALCPTTLAPYYLR |  | |  |  |  |
|  |  |  | 3517.89 | LPPGVDPAAVTSALSPEGVLSIQAAPASAQAPPPAAAK |  | |  |  |  |
|  |  |  | 3837.05 | YRLPPGVDPAAVTSALSPEGVLSIQAAPASAQAPPPAAAK |  | |  |  |  |
|  |  |  |  |  |  |  |  |  |  |
| 739 | 18 / 50 | 475 | 750.40 | FLEDVK | Alpha-1-antitrypsin | | A1AT_HUMAN | 44324.55 | 5.37 |
|  |  |  | 795.41 | SPLFMGK |  |  |  |  |  |
|  |  |  | 922.43 | FLENEDR |  | |  |  |  |
|  |  |  | 1078.53 | *FLENEDRR |  | |  |  |  |
|  |  |  | 1090.57 | WERPFEVK |  | |  |  |  |
|  |  |  | 1263.60 | LGMFNIQHCK |  | |  |  |  |
|  |  |  | 1275.68 | *GKWERPFEVK |  | |  |  |  |
|  |  |  | 1576.84 | DTVFALVNYIFFK |  | |  |  |  |
|  |  |  | 1641.86 | *ITPNLAEFAFSLYR |  | |  |  |  |
|  |  |  | 1779.77 | TDTSHHDQDHPTFNK |  | |  |  |  |
|  |  |  | 1803.96 | LQHLENELTHDIITK |  | |  |  |  |
|  |  |  | 1833.92 | VFSNGADLSGVTEEAPLK |  | |  |  |  |
|  |  |  | 1871.97 | *FNKPFVFLMIEQNTK |  | |  |  |  |
|  |  |  | 1891.86 | *DTEEEDFHVDQVTTVK |  | |  |  |  |
|  |  |  | 2057.95 | LYHSEAFTVNFGDTEEAK |  | |  |  |  |
|  |  |  | 2186.04 | LYHSEAFTVNFGDTEEAKK |  | |  |  |  |
|  |  |  | 2291.13 | GTEAAGAMFLEAIPMSIPPEVK |  | |  |  |  |
|  |  |  | 2574.34 | *TLNQPDSQLQLTTGNGLFLSEGLK |  | |  |  |  |
| 878 | 22 / 46 | 446 | 1081.57 | LLQDFFNGK | Heat shock cognate 71 kDa protein | | HSP7C_HUMAN | 70766.90 | 5.37 |
|  |  |  | 1197.66 | FELTGIPPAPR |  |  |  |  |  |
|  |  |  | 1199.67 | DAGTIAGLNVLR |  |  |  |  |  |
|  |  |  | 1228.63 | VEIIANDQGNR |  | |  |  |  |
|  |  |  | 1251.62 | MVNHFIAEFK |  | |  |  |  |
|  |  |  | 1253.62 | *FEELNADLFR |  | |  |  |  |
|  |  |  | 1268.66 | MKEIAEAYLGK |  | |  |  |  |
|  |  |  | 1319.59 | NSLESYAFNMK |  | |  |  |  |
|  |  |  | 1426.66 | RFDDAVVQSDMK |  | |  |  |  |
|  |  |  | 1480.75 | ARFEELNADLFR |  | |  |  |  |
|  |  |  | 1481.81 | SQIHDIVLVGGSTR |  | |  |  |  |
|  |  |  | 1487.70 | *TTPSYVAFTDTER |  | |  |  |  |
|  |  |  | 1632.78 | SFYPEEVSSMVLTK |  | |  |  |  |
|  |  |  | 1659.90 | IINEPTAAAIAYGLDK |  | |  |  |  |
|  |  |  | 1665.79 | NQVAMNPTNTVFDAK |  | |  |  |  |
|  |  |  | 1669.83 | HWPFMVVNDAGRPK |  | |  |  |  |
|  |  |  | 1691.73 | STAGDTHLGGEDFDNR |  | |  |  |  |
|  |  |  | 1745.81 | NQTAEKEEFEHQQK |  | |  |  |  |
|  |  |  | 1982.00 | *TVTNAVVTVPAYFNDSQR |  | |  |  |  |
|  |  |  | 2260.15 | SINPDEAVAYGAAVQAAILSGDK |  | |  |  |  |
|  |  |  | 2263.12 | GPAVGIDLGTTYSCVGVFQHGK |  | |  |  |  |
|  |  |  | 2774.33 | QTQTFTTYSDNQPGVLIQVYEGER |  | |  |  |  |
| 890 | 10 / 37 | 222 | 909.42 | AANWYER | NADH dehydrogenase [ubiquinone] iron-sulfur protein 3, mitochondrial | | NDUS3_HUMAN | 26414.92 | 5.48 |
|  |  |  | 1295.66 | DFPLSGYVELR |  |  |  |  |  |
|  |  |  | 1366.77 | FEIVYNLLSLR |  |  |  |  |  |
|  |  |  | 1385.76 | SLVDLTAVDVPTR |  |  |  |  |  |
|  |  |  | 1486.79 | *VVAEPVELAQEFR |  | |  |  |  |
|  |  |  | 1512.79 | ESAGADTRPTVRPR |  | |  |  |  |
|  |  |  | 1551.76 | *ILTDYGFEGHPFR |  | |  |  |  |
|  |  |  | 1707.86 | RILTDYGFEGHPFR |  | |  |  |  |
|  |  |  | 1740.84 | FDLNSPWEAFPVYR |  | |  |  |  |
|  |  |  | 1868.93 | KFDLNSPWEAFPVYR |  | |  |  |  |
| 907 | 31 / 54 | 742 | 728.44 | NINIVR | Acolitase hydratase, mitochondrial | | ACON_HUMAN | 82425.78 | 6.85 |
|  |  |  | 772.49 | LTIQGLK |  |  |  |  |  |
|  |  |  | 922.47 | EHAALEPR |  | |  |  |  |
|  |  |  | 935.49 | *DGYAQILR |  | |  |  |  |
|  |  |  | 985.51 | *EGWPLDIR |  | |  |  |  |
|  |  |  | 1067.55 | NTIVTSYNR |  | |  |  |  |
|  |  |  | 1102.61 | LQLLEPFDK |  | |  |  |  |
|  |  |  | 1170.68 | LNRPLTLSEK |  | |  |  |  |
|  |  |  | 1219.63 | LTGSLSGWSSPK |  | |  |  |  |
|  |  |  | 1264.61 | EDIANLADEFK |  | |  |  |  |
|  |  |  | 1326.79 | RLNRPLTLSEK |  | |  |  |  |
|  |  |  | 1463.75 | *SQFTITPGSEQIR |  | |  |  |  |
|  |  |  | 1500.77 | FRLEAPDADELPK |  | |  |  |  |
|  |  |  | 1556.75 | CTTDHISAAGPWLK |  | |  |  |  |
|  |  |  | 1557.71 | FNPETDYLTGTDGK |  | |  |  |  |
|  |  |  | 1588.84 | LQLLEPFDKWDGK |  | |  |  |  |
|  |  |  | 1599.74 | DSSGQHVDVSPTSQR |  | |  |  |  |
|  |  |  | 1601.79 | *NAVTQEFGPVPDTAR |  | |  |  |  |
|  |  |  | 1667.77 | *WVVIGDENYGEGSSR |  | |  |  |  |
|  |  |  | 1715.77 | GEFDPGQDTYQHPPK |  | |  |  |  |
|  |  |  | 1753.88 | DINQEVYNFLATAGAK |  | |  |  |  |
|  |  |  | 1762.90 | QGLLPLTFADPADYNK |  | |  |  |  |
|  |  |  | 1841.90 | *IVYGHLDDPASQEIER |  | |  |  |  |
|  |  |  | 1861.81 | VGLIGSCTNSSYEDMGR |  | |  |  |  |
|  |  |  | 2172.03 | DLGGIVLANACGPCIGQWDR |  | |  |  |  |
|  |  |  | 2219.18 | GHLDNISNNLLIGAINIENGK |  | |  |  |  |
|  |  |  | 2260.14 | VAVPSTIHCDHLIEAQVGGEK |  | |  |  |  |
|  |  |  | 2351.10 | VAMSHFEPNEYIHYDLLEK |  | |  |  |  |
|  |  |  | 2376.12 | VAMQDATAQMAMLQFISSGLSK |  | |  |  |  |
|  |  |  | 2780.45 | NDANPETHAFVTSPEIVTALAIAGTLK |  | |  |  |  |
|  |  |  | 2925.43 | HPNGTQETILLNHTFNETQIEWFR |  | |  |  |  |
|  |  |  |  |  | |  |  |  |  |

^a^Number of matched peptides

^b^Percentage of coverage of full length protein by tryptic peptides
