# Supplementary material for: Cardiac protein changes in ischaemic and dilated cardiomyopathy: a proteomic study of human left ventricular tissue
Source: J Cell Mol Med. 2012 Sep 26;16(10):2471–86. doi: 10.1111/j.1582-4934.2012.01565.x (PMC3823441; doi:10.1111/j.1582-4934.2012.01565.x)
Supplement: Supplementary file 2 [file jcmm0016-2471-SD2.doc]

**Supplementary table 2:** Additional data on MS protein identification of DCM spots with differential expression by MALDI-MS. Peptides submitted to MS/MS, as an additional analysis that led to the same protein identification, are marked with an asterisk.

| **Spot** | **N^a^ / %^b^** | **Mascot Score** | **Peptides identified by MS** | | **Identified protein** | **Accession number** | **Expected Mw** | **Expected pI** |
| --- | --- | --- | --- | --- | --- | --- | --- | --- |
|  |  |  | **M+H** | **Sequence** |  |  |  |  |
| 23 | 12 / 31 | 428 | 713.43 | *ELKPAR | ATP synthase subunit gamma, mitochondrial (isoform heart) | ATPG_HUMAN | 32880.96 | 9.31 |
|  |  |  | 864.49 | *LTLTFNR |  |  |  |  |
|  |  |  | 1076.57 | EVMLVGIGDK |  |  |  |  |
|  |  |  | 1095.49 | ESTTSEQSAR |  |  |  |  |
|  |  |  | 1096.61 | *HLLIGVSSDR |  |  |  |  |
|  |  |  | 1213.64 | *GLCGAIHSSIAK |  |  |  |  |
|  |  |  | 1224.71 | KHLLIGVSSDR |  |  |  |  |
|  |  |  | 1292.66 | *THSDQFLVAFK |  |  |  |  |
|  |  |  | 1314.72 | ELIEIISGAAALD |  |  |  |  |
|  |  |  | 1326.73 | *IYGLGSLALYEK |  |  |  |  |
|  |  |  | 1329.76 | EVMLVGIGDKIR |  |  |  |  |
|  |  |  | 2291.23 | HLLIGVSSDRGLCGAIHSSIAK |  |  |  |  |
| 29 | 11 / 65 | 381 | 857.41 | YVDMSVK | ATP synthase subunit O, mitochondrial | ATPO_HUMAN | 20875.49 | 9.81 |
|  |  |  | 861.47 | SLNDITAK |  |  |  |  |
|  |  |  | 1106.62 | *SFLSQGQVLK |  |  |  |  |
|  |  |  | 1115.61 | QNKLEQVEK |  |  |  |  |
|  |  |  | 1145.58 | YATALYSAASK |  |  |  |  |
|  |  |  | 1160.67 | *VAASVLNPYVK |  |  |  |  |
|  |  |  | 1274.68 | TDPSILGGMIVR |  |  |  |  |
|  |  |  | 1582.91 | *LVRPPVQVYGIEGR |  |  |  |  |
|  |  |  | 1873.02 | *FSPLTTNLINLLAENGR |  |  |  |  |
|  |  |  | 2083.99 | LSNTQGVVSAFSTMMSVHR |  |  |  |  |
|  |  |  | 2318.14 | GEVPCTVTSASPLEEATLSELK |  |  |  |  |
| 66 | 22 / 48 | 578 | 707.38 | QAVAYR | ATP synthase subunit alpha, mitochondrial | ATPA_HUMAN | 55209.32 | 8.28 |
|  |  |  | 716.46 | LTELLK |  |  |  |  |
|  |  |  | 723.45 | APGIIPR |  |  |  |  |
|  |  |  | 789.42 | VGSAAQTR |  |  |  |  |
|  |  |  | 815.46 | ELIIGDR |  |  |  |  |
|  |  |  | 876.50 | QMSLLLR |  |  |  |  |
|  |  |  | 892.49 | *LELAQYR |  |  |  |  |
|  |  |  | 1000.58 | VLSIGDGIAR |  |  |  |  |
|  |  |  | 1026.59 | *AVDSLVPIGR |  |  |  |  |
|  |  |  | 1036.51 | RLTDADAMK |  |  |  |  |
|  |  |  | 1316.74 | TSIAIDTIINQK |  |  |  |  |
|  |  |  | 1327.71 | LYCIYVAIGQK |  |  |  |  |
|  |  |  | 1438.85 | GIRPAINVGLSVSR |  |  |  |  |
|  |  |  | 1439.67 | TGTAEMSSILEER |  |  |  |  |
|  |  |  | 1553.74 | *EAYPGDVFYLHSR |  |  |  |  |
|  |  |  | 1575.79 | *ILGADTSVDLEETGR |  |  |  |  |
|  |  |  | 1624.89 | *TGAIVDVPVGEELLGR |  |  |  |  |
|  |  |  | 1683.79 | NVQAEEMVEFSSGLK |  |  |  |  |
|  |  |  | 2120.03 | GMSLNLEPDNVGVVVFGNDK |  |  |  |  |
|  |  |  | 2325.15 | QGQYSPMAIEEQVAVIYAGVR |  |  |  |  |
|  |  |  | 2338.17 | *EVAAFAQFGSDLDAATQQLLSR |  |  |  |  |
|  |  |  | 2367.26 | *FENAFLSHVVSQHQALLGTIR |  |  |  |  |
| 69 | 15 / 34 | 172 | 707.38 | QAVAYR | ATP synthase subunit alpha, mitochondrial | ATPA_HUMAN | 55209.32 | 8.28 |
|  |  |  | 723.45 | APGIIPR |  |  |  |  |
|  |  |  | 815.47 | GPIGSKTR |  |  |  |  |
|  |  |  | 876.50 | QMSLLLR |  |  |  |  |
|  |  |  | 892.49 | LELAQYR |  |  |  |  |
|  |  |  | 1000.58 | VLSIGDGIAR |  |  |  |  |
|  |  |  | 1026.59 | AVDSLVPIGR |  |  |  |  |
|  |  |  | 1438.85 | GIRPAINVGLSVSR |  |  |  |  |
|  |  |  | 1553.74 | *EAYPGDVFYLHSR |  |  |  |  |
|  |  |  | 1575.79 | ILGADTSVDLEETGR |  |  |  |  |
|  |  |  | 1624.89 | *TGAIVDVPVGEELLGR |  |  |  |  |
|  |  |  | 1683.79 | NVQAEEMVEFSSGLK |  |  |  |  |
|  |  |  | 2325.15 | QGQYSPMAIEEQVAVIYAGVR |  |  |  |  |
|  |  |  | 2338.17 | EVAAFAQFGSDLDAATQQLLSR |  |  |  |  |
|  |  |  | 2367.26 | FENAFLSHVVSQHQALLGTIR |  |  |  |  |
| 78 | 13 / 25 | 183 | 722.35 | DLDGFR | UTP-glucose-1-phosphate urydylyltransferase | UGPA_HUMAN | 56809.06 | 8.15 |
|  |  |  | 749.31 | YNHCR |  |  |  |  |
|  |  |  | 793.42 | VQDYLR |  |  |  |  |
|  |  |  | 850.44 | DLDGFRK |  |  |  |  |
|  |  |  | 1028.52 | IYTFNQSR |  |  |  |  |
|  |  |  | 1265.70 | GTVIIIANHGDR |  |  |  |  |
|  |  |  | 1332.69 | *SFENSLGINVPR |  |  |  |  |
|  |  |  | 1575.82 | SFENSLGINVPRSR |  |  |  |  |
|  |  |  | 1600.80 | ILTTASSHEFEHTK |  |  |  |  |
|  |  |  | 1682.78 | AMSQDGASQFQEVIR |  |  |  |  |
|  |  |  | 1699.87 | *IQRPPEDSIQPYEK |  |  |  |  |
|  |  |  | 1844.94 | LQEQNAIDMEIIVNAK |  |  |  |  |
|  |  |  | 2156.10 | NENTFLDLTVQQIEHLNK |  |  |  |  |
| 86 | 25 / 65 | 778 | 731.43 | ELASALK | Annexin A2 | ANXA2_HUMAN | 38472.85 | 7.56 |
|  |  |  | 772.35 | LYDSMK |  |  |  |  |
|  |  |  | 837.40 | SEVDMLK |  |  |  |  |
|  |  |  | 880.44 | DLYDAGVK |  |  |  |  |
|  |  |  | 976.48 | VYKEMYK |  |  |  |  |
|  |  |  | 1036.54 | DLYDAGVKR |  |  |  |  |
|  |  |  | 1051.52 | WISIMTER |  |  |  |  |
|  |  |  | 1086.49 | *AYTNFDAER |  |  |  |  |
|  |  |  | 1111.55 | *QDIAFAYQR |  |  |  |  |
|  |  |  | 1222.60 | TPAQYDASELK |  |  |  |  |
|  |  |  | 1225.57 | DIISDTSGDFR |  |  |  |  |
|  |  |  | 1244.62 | *TNQELQEINR |  |  |  |  |
|  |  |  | 1421.69 | *SLYYYIQQDTK |  |  |  |  |
|  |  |  | 1476.67 | *SYSPYDMLESIR |  |  |  |  |
|  |  |  | 1542.85 | *GVDEVTIVNILTNR |  |  |  |  |
|  |  |  | 1604.76 | SYSPYDMLESIRK |  |  |  |  |
|  |  |  | 1632.84 | GTDVPKWISIMTER |  |  |  |  |
|  |  |  | 1650.98 | *SALSGHLETVILGLLK |  |  |  |  |
|  |  |  | 1771.99 | TKGVDEVTIVNILTNR |  |  |  |  |
|  |  |  | 1777.86 | GLGTDEDSLIEIICSR |  |  |  |  |
|  |  |  | 1811.87 | TDLEKDIISDTSGDFR |  |  |  |  |
|  |  |  | 1844.90 | *LSLEGDHSTPPSAYGSVK |  |  |  |  |
|  |  |  | 1908.88 | AEDGSVIDYELIDQDAR |  |  |  |  |
|  |  |  | 2064.98 | RAEDGSVIDYELIDQDAR |  |  |  |  |
|  |  |  | 2155.07 | AYTNFDAERDALNIETAIK |  |  |  |  |
| 87 | 19 / 62 | 686 | 772.35 | LYDSMK | Annexin A2 | ANXA2_HUMAN | 38472.85 | 7.56 |
|  |  |  | 880.44 | DLYDAGVK |  |  |  |  |
|  |  |  | 1051.52 | WISIMTER |  |  |  |  |
|  |  |  | 1086.49 | *AYTNFDAER |  |  |  |  |
|  |  |  | 1111.55 | *QDIAFAYQR |  |  |  |  |
|  |  |  | 1222.60 | TPAQYDASELK |  |  |  |  |
|  |  |  | 1225.57 | DIISDTSGDFR |  |  |  |  |
|  |  |  | 1244.62 | *TNQELQEINR |  |  |  |  |
|  |  |  | 1421.69 | *SLYYYIQQDTK |  |  |  |  |
|  |  |  | 1476.67 | *SYSPYDMLESIR |  |  |  |  |
|  |  |  | 1542.85 | *GVDEVTIVNILTNR |  |  |  |  |
|  |  |  | 1632.84 | GTDVPKWISIMTER |  |  |  |  |
|  |  |  | 1650.98 | SALSGHLETVILGLLK |  |  |  |  |
|  |  |  | 1777.86 | GLGTDEDSLIEIICSR |  |  |  |  |
|  |  |  | 1811.87 | TDLEKDIISDTSGDFR |  |  |  |  |
|  |  |  | 1844.90 | LSLEGDHSTPPSAYGSVK |  |  |  |  |
|  |  |  | 1908.88 | AEDGSVIDYELIDQDAR |  |  |  |  |
|  |  |  | 2064.98 | RAEDGSVIDYELIDQDAR |  |  |  |  |
|  |  |  | 2838.42 | GDLENAFLNLVQCIQNKPLYFADR |  |  |  |  |
| 94 | 16 / 51 | 421 | 714.46 | KLNIAR | Acetyl-CoA acetyltransferase, mitochondrial | THIL_HUMAN | 41386.02 | 8.16 |
|  |  |  | 865.44 | GSTPYGGVK |  |  |  |  |
|  |  |  | 883.54 | LNVTPLAR |  |  |  |  |
|  |  |  | 968.43 | EDEEYKR |  |  |  |  |
|  |  |  | 973.57 | *EVVIVSATR |  |  |  |  |
|  |  |  | 1024.49 | DGLTDVYNK |  |  |  |  |
|  |  |  | 1039.64 | RLNVTPLAR |  |  |  |  |
|  |  |  | 1199.70 | LGSIAIQGAIEK |  |  |  |  |
|  |  |  | 1402.79 | FGNEVIPVTVTVK |  |  |  |  |
|  |  |  | 1544.70 | *NEQDAYAINSYTR |  |  |  |  |
|  |  |  | 1701.98 | *TPIGSFLGSLSLLPATK |  |  |  |  |
|  |  |  | 1893.88 | *EAYMGNVLQGGEGQAPTR |  |  |  |  |
|  |  |  | 1922.99 | VNINGGAVSLGHPIGMSGAR |  |  |  |  |
|  |  |  | 2282.09 | QGEYGLASICNGGGGASAMLIQK |  |  |  |  |
|  |  |  | 2777.36 | ENGTVTAANASTLNDGAAALVLMTADAAK |  |  |  |  |
|  |  |  | 2834.50 | IVAFADAAVEPIDFPIAPVYAASMVLK |  |  |  |  |
| 107 | 6 / 25 | 110 | 811.41 | LTGMAFR | Glyceraldehyde-3-phosphate dehydrogenase | G3P_HUMAN | 35922.02 | 8.58 |
|  |  |  | 1530.79 | VPTANVSVVDLTCR |  |  |  |  |
|  |  |  | 1661.90 | VIPELNGKLTGMAFR |  |  |  |  |
|  |  |  | 1763.80 | *LISWYDNEFGYSNR |  |  |  |  |
|  |  |  | 2245.10 | VIISAPSADAPMFVMGVNHEK |  |  |  |  |
|  |  |  | 2293.03 | WGDAGAEYVVESTGVFTTMEK |  |  |  |  |
| 141 | 8 / 51 | 226 | 746.39 | REQWK | NADH dehydrogenase [ubiquinone] 1 beta subcomplex subunit 9 | NDUB9_HUMAN | 21699.72 | 8.59 |
|  |  |  | 976.44 | YFACLMR |  |  |  |  |
|  |  |  | 1121.50 | AMYPDYFAK |  |  |  |  |
|  |  |  | 1214.57 | HLESWCVQR |  |  |  |  |
|  |  |  | 1258.54 | *EAEEEFWYR |  |  |  |  |
|  |  |  | 1797.79 | VPEWCLDDWHPSEK |  |  |  |  |
|  |  |  | 2101.09 | *QLQEETPPGGPLTEALPPAR |  |  |  |  |
|  |  |  | 2176.01 | QHPQPYIFPDSPGGTSYER |  |  |  |  |
| 196 | 8 / 15 | 84 | 704,43 | VTGATKK | Dihydrolipoyl dehydrogenase, mitochondrial | DLDH_HUMAN | 50174.62 | 6.50 |
|  |  |  | 909,46 | *FPFAANSR |  |  |  |  |
|  |  |  | 912,54 | GRIPVNTR |  |  |  |  |
|  |  |  | 936,45 | GIEMSEVR |  |  |  |  |
|  |  |  | 972,53 | VVHVNGYGK |  |  |  |  |
|  |  |  | 1970,09 | MVVIGAGVIGVELGSVWQR |  |  |  |  |
|  |  |  | 1986,08 | MVVIGAGVIGVELGSVWQR |  |  |  |  |
|  |  |  | 1995,07 | IPNIYAIGDVVAGPMLAHK |  |  |  |  |
| 197 | 11 / 24 | 159 | 744.43 | LVEDLR | Glutamate dehydrogenase 1, mitochondrial | DHE3_HUMAN | 56008.68 | 6.71 |
|  |  |  | 963.53 | YNLGLDLR |  |  |  |  |
|  |  |  | 1016.45 | MVEGFFDR |  |  |  |  |
|  |  |  | 1059.53 | *NLNHVSYGR |  |  |  |  |
|  |  |  | 1196.68 | LQHGSILGFPK |  |  |  |  |
|  |  |  | 1425.63 | DDGSWEVIEGYR |  |  |  |  |
|  |  |  | 1507.72 | DIVHSGLAYTMER |  |  |  |  |
|  |  |  | 1737.89 | *HGGTIPIVPTAEFQDR |  |  |  |  |
|  |  |  | 1764.89 | TFVVQGFGNVGLHSMR |  |  |  |  |
|  |  |  | 1931.92 | GFIGPGIDVPAPDMSTGER |  |  |  |  |
|  |  |  | 1936.91 | DSNYHLLMSVQESLER |  |  |  |  |
| 204 | 14 / 38 | 289 | 722.38 | LTADFR | Aspartate aminotransferase, cytoplasmic | AATC_HUMAN | 46116.31 | 6.57 |
|  |  |  | 731.38 | DAWAIR |  |  |  |  |
|  |  |  | 862.48 | IGADFLAR |  |  |  |  |
|  |  |  | 948.53 | VNLGVGAYR |  |  |  |  |
|  |  |  | 1012.48 | *NFGLYNER |  |  |  |  |
|  |  |  | 1055.60 | HIYLLPSGR |  |  |  |  |
|  |  |  | 1076.62 | KVNLGVGAYR |  |  |  |  |
|  |  |  | 1271.71 | VGGVQSLGGTGALR |  |  |  |  |
|  |  |  | 1297.66 | ITWSNPPAQGAR |  |  |  |  |
|  |  |  | 1565.78 | TDDCHPWVLPVVK |  |  |  |  |
|  |  |  | 1646.83 | EPESILQVLSQMEK |  |  |  |  |
|  |  |  | 2024.12 | APPSVFAEVPQAQPVLVFK |  |  |  |  |
|  |  |  | 2313.10 | FLFPFFDSAYQGFASGNLER |  |  |  |  |
|  |  |  | 2399.24 | *IANDNSLNHEYLPILGLAEFR |  |  |  |  |
|  | 16 / 45 | 259 | 704.41 | GVPLYR | Alpha-enolase | ENOA_HUMAN | 47037.77 | 6.99 |
|  |  |  | 766.37 | EIFDSR |  |  |  |  |
|  |  |  | 806.45 | YNQLLR |  |  |  |  |
|  |  |  | 810.45 | AVEHINK |  |  |  |  |
|  |  |  | 1143.62 | IGAEVYHNLK |  |  |  |  |
|  |  |  | 1425.73 | YISPDQLADLYK |  |  |  |  |
|  |  |  | 1540.78 | VVIGMDVAASEFFR |  |  |  |  |
|  |  |  | 1541.76 | LAQANGWGVMVSHR |  |  |  |  |
|  |  |  | 1556.78 | VVIGMDVAASEFFR |  |  |  |  |
|  |  |  | 1633.82 | VNQIGSVTESLQACK |  |  |  |  |
|  |  |  | 1646.83 | EAMRIGAEVYHNLK |  |  |  |  |
|  |  |  | 1804.94 | AAVPSGASTGIYEALELR |  |  |  |  |
|  |  |  | 1939.98 | LAMQEFMILPVGAANFR |  |  |  |  |
|  |  |  | 1960.92 | DATNVGDEGGFAPNILENK |  |  |  |  |
|  |  |  | 2033.05 | FTASAGIQVVGDDLTVTNPK |  |  |  |  |
|  |  |  | 3011.57 | HIADLAGNSEVILPVPAFNVINGGSHAGNK |  |  |  |  |
| 211 | 17 / 51 | 543 | 734.42 | AAQLGFK | Dihydrolipoyl dehydrogenase, mitochondrial | DLDH_HUMAN | 50174.62 | 6.50 |
|  |  |  | 909.46 | *FPFAANSR |  |  |  |  |
|  |  |  | 912.54 | GRIPVNTR |  |  |  |  |
|  |  |  | 936.45 | GIEMSEVR |  |  |  |  |
|  |  |  | 972.53 | VVHVNGYGK |  |  |  |  |
|  |  |  | 1007.52 | EANLAASFGK |  |  |  |  |
|  |  |  | 1127.66 | *ALTGGIAHLFK |  |  |  |  |
|  |  |  | 1524.74 | *VCHAHPTLSEAFR |  |  |  |  |
|  |  |  | 1567.83 | *NLGLEELGIELDPR |  |  |  |  |
|  |  |  | 1581.76 | *SEEQLKEEGIEYK |  |  |  |  |
|  |  |  | 1717.90 | *AEVITCDVLLVCIGR |  |  |  |  |
|  |  |  | 1819.87 | NETLGGTCLNVGCIPSK |  |  |  |  |
|  |  |  | 1986.08 | MVVIGAGVIGVELGSVWQR |  |  |  |  |
|  |  |  | 1995.07 | IPNIYAIGDVVAGPMLAHK |  |  |  |  |
|  |  |  | 2530.29 | LGADVTAVEFLGHVGGVGIDMEISK |  |  |  |  |
|  |  |  | 3359.78 | NILIATGSEVTPFPGITIDEDTIVSSTGALSLK |  |  |  |  |
|  |  |  | 3370.64 | VLGAHILGPGAGEMVNEAALALEYGASCEDIAR |  |  |  |  |
| 252 | 13 / 36 | 295 | 716.38 | *WQVQR | Alcohol dehydrogenase [NADP+] | AK1A1_HUMAN | 36441.85 | 6.35 |
|  |  |  | 897.55 | *SPAQILLR |  |  |  |  |
|  |  |  | 928.49 | *YALSVGYR |  |  |  |  |
|  |  |  | 1131.62 | MPLIGLGTWK |  |  |  |  |
|  |  |  | 1235.67 | YIVPMLTVDGK |  |  |  |  |
|  |  |  | 1251.67 | YIVPMLTVDGK |  |  |  |  |
|  |  |  | 1299.64 | HHPEDVEPALR |  |  |  |  |
|  |  |  | 1375.76 | *AVPREELFVTSK |  |  |  |  |
|  |  |  | 1427.74 | HHPEDVEPALRK |  |  |  |  |
|  |  |  | 1475.80 | GLVQALGLSNFNSR |  |  |  |  |
|  |  |  | 1492.67 | VFDFTFSPEEMK |  |  |  |  |
|  |  |  | 1505.67 | *DAGHPLYPFNDPY |  |  |  |  |
|  |  |  | 1551.76 | *GLEVTAYSPLGSSDR |  |  |  |  |
| 312 | 10 / 62 | 198 | 700.44 | TIPITR | Alpha-crystallin B chain | CRYAB_HUMAN | 20158.91 | 6.76 |
|  |  |  | 772.39 | QVSGPER |  |  |  |  |
|  |  |  | 921.50 | FSVNLDVK |  |  |  |  |
|  |  |  | 986.49 | HFSPEELK |  |  |  |  |
|  |  |  | 1088.51 | *QDEHGFISR |  |  |  |  |
|  |  |  | 1165.66 | VLGDVIEVHGK |  |  |  |  |
|  |  |  | 1374.71 | *RPFFPFHSPSR |  |  |  |  |
|  |  |  | 1388.73 | MDIAIHHPWIR |  |  |  |  |
|  |  |  | 1512.68 | APSWFDTGLSEMR |  |  |  |  |
|  |  |  | 2624.38 | IPADVDPLTITSSLSSDGVLTVNGPR |  |  |  |  |
| 330 | 4 / 7 | 89 | 781.37 | ECGFIR | THAP domain-containing protein 4 | THAP4_HUMAN | 62889.85 | 9.39 |
|  |  |  | 1108.57 | *EPHVEQITR |  |  |  |  |
|  |  |  | 1446.71 | KPMHRECGFIR |  |  |  |  |
|  |  |  | 2677.30 | LEQTVSMATTTQPMTQHLHVTYK |  |  |  |  |
| 339 | 9 / 54 | 425 | 933.54 | VNLAELFK | Peroxiredoxin-5 | PRDX5_HUMAN | 17030.75 | 6.73 |
|  |  |  | 1089.59 | LLADPTGAFGK |  |  |  |  |
|  |  |  | 1238.64 | FSMVVQDGIVK |  |  |  |  |
|  |  |  | 1394.75 | RFSMVVQDGIVK |  |  |  |  |
|  |  |  | 1539.82 | *THLPGFVEQAEALK |  |  |  |  |
|  |  |  | 1593.81 | GVLFGVPGAFTPGCSK |  |  |  |  |
|  |  |  | 1827.91 | *VGDAIPAVEVFEGEPGNK |  |  |  |  |
|  |  |  | 1906.98 | *ETDLLLDDSLVSIFGNR |  |  |  |  |
|  |  |  | 2264.11 | GVQVVACLSVNDAFVTGEWGR |  |  |  |  |
|  | 8 / 48 | 177 | 705.38 | ALSTGEK | Peptidyl-prolyl cis-trans isomerase | PPIA_HUMAN | 17881.30 | 7.82 |
|  |  |  | 737.36 | TAENFR |  |  |  |  |
|  |  |  | 763.33 | GSCFHR |  |  |  |  |
|  |  |  | 1055.54 | VSFELFADK |  |  |  |  |
|  |  |  | 1154.57 | FEDENFILK |  |  |  |  |
|  |  |  | 1310.57 | EGMNIVEAMER |  |  |  |  |
|  |  |  | 1614.74 | IIPGFMCQGGDFTR |  |  |  |  |
|  |  |  | 1946.00 | *VNPTVFFDIAVDGEPLGR |  |  |  |  |
| 366 | 25 / 36 | 534 | 717.38 | ELAEQK | Mitochondrial inner membrane protein | IMMT_HUMAN | 83677.91 | 6.08 |
|  |  |  | 748.44 | FVNQLK |  |  |  |  |
|  |  |  | 755.41 | FYAVQK |  |  |  |  |
|  |  |  | 758.42 | IDQLNR |  |  |  |  |
|  |  |  | 762.40 | ALEHHR |  |  |  |  |
|  |  |  | 773.45 | KIEEVR |  |  |  |  |
|  |  |  | 803.43 | SLEDALR |  |  |  |  |
|  |  |  | 847.38 | WDSHFR |  |  |  |  |
|  |  |  | 914.52 | RIDQLNR |  |  |  |  |
|  |  |  | 1015.57 | RVAQDWLK |  |  |  |  |
|  |  |  | 1053.52 | GVYSEETLR |  |  |  |  |
|  |  |  | 1075.50 | SEIQAEQDR |  |  |  |  |
|  |  |  | 1119.57 | QAAAHTDHLR |  |  |  |  |
|  |  |  | 1149.59 | *LSEQELQFR |  |  |  |  |
|  |  |  | 1210.56 | SEFEQNLSEK |  |  |  |  |
|  |  |  | 1522.87 | KAHQLWLSVEALK |  |  |  |  |
|  |  |  | 1525.80 | LHNMIVDLDNVVK |  |  |  |  |
|  |  |  | 1527.83 | *VVSQYHELVVQAR |  |  |  |  |
|  |  |  | 1662.86 | LSTDDLNSLIAHAHR |  |  |  |  |
|  |  |  | 1823.89 | *GIEQAVQSHAVAEEEAR |  |  |  |  |
|  |  |  | 1872.00 | TSSAETPTIPLGSAVEAIK |  |  |  |  |
|  |  |  | 1881.86 | ACQLSGVTAAAQSCLCGK |  |  |  |  |
|  |  |  | 2153.06 | LLSYASYCIEHGDLELAAK |  |  |  |  |
|  |  |  | 2198.07 | *LSQEQVDNFTLDINTAYAR |  |  |  |  |
|  |  |  | 2506.19 | ANCSDNEFTQALTAAIPPESLTR |  |  |  |  |
|  | 17 / 27 | 232 | 788.43 | VSAQEVR | Ezrin | EZRI_HUMAN | 69281.61 | 5.95 |
|  |  |  | 823.51 | PKPINVR |  |  |  |  |
|  |  |  | 894.54 | LFFLQVK |  |  |  |  |
|  |  |  | 914.53 | IALLEEAR |  |  |  |  |
|  |  |  | 976.55 | QLFDQVVK |  |  |  |  |
|  |  |  | 987.51 | ALQLEEER |  |  |  |  |
|  |  |  | 1002.52 | ELSEQIQR |  |  |  |  |
|  |  |  | 1104.58 | *IGFPWSEIR |  |  |  |  |
|  |  |  | 1120.56 | QRIDEFEAL |  |  |  |  |
|  |  |  | 1175.61 | IQVWHAEHR |  |  |  |  |
|  |  |  | 1182.59 | *APDFVFYAPR |  |  |  |  |
|  |  |  | 1310.69 | *KAPDFVFYAPR |  |  |  |  |
|  |  |  | 1445.80 | QLLTLSSELSQAR |  |  |  |  |
|  |  |  | 1525.78 | GMLKDNAMLEYLK |  |  |  |  |
|  |  |  | 1651.82 | SQEQLAAELAEYTAK |  |  |  |  |
|  |  |  | 1669.80 | EVWYFGLHYVDNK |  |  |  |  |
|  |  |  | 2082.01 | VTTMDAELEFAIQPNTTGK |  |  |  |  |
| 447 | 6 / 26 | 221 | 1214.62 | LGTQPYFFNK | Metaxin-2 | MTX2_HUMAN | 29763.19 | 5.90 |
|  |  |  | 1236.56 | IEQHYFEDR |  |  |  |  |
|  |  |  | 1257.60 | NYSNLLAFCR |  |  |  |  |
|  |  |  | 1392.67 | *RIEQHYFEDR |  |  |  |  |
|  |  |  | 2278.04 | *TLDQVLEDVDQCCQALSQR |  |  |  |  |
|  |  |  | 2406.35 | *VPFIHVGNQVVSELGPIVQFVK |  |  |  |  |
| 489 | 6 / 28 | 61 | 816.51 | GKPLFVR | Cytochrome b-c1 complex subunit Rieske, mitochondrial | UCRI_HUMAN | 49128.60 | 5.43 |
|  |  |  | 1012.47 | VPDFSEYR |  |  |  |  |
|  |  |  | 1060.60 | RLEVLDSTK |  |  |  |  |
|  |  |  | 1614.83 | EIEQEAAVELSQLR |  |  |  |  |
|  |  |  | 2032.11 | KGFSYLVTGVTTVGVAYAAK |  |  |  |  |
|  |  |  | 2126.08 | NAVTQFVSSMSASADVLALAK |  |  |  |  |
| 540 | 23 / 40 | 898 | 748.34 | VCQGER | Stress-70 protein, mitochondrial | GRP75_HUMAN | 68759.00 | 5.44 |
|  |  |  | 817.42 | TIAPCQK |  |  |  |  |
|  |  |  | 863.40 | DNMALQR |  |  |  |  |
|  |  |  | 938.43 | YAEEDRR |  |  |  |  |
|  |  |  | 958.50 | VLENAEGAR |  |  |  |  |
|  |  |  | 960.55 | DIKNVPFK |  |  |  |  |
|  |  |  | 1148.55 | KDSETGENIR |  |  |  |  |
|  |  |  | 1149.55 | RYDDPEVQK |  |  |  |  |
|  |  |  | 1242.68 | DAGQISGLNVLR |  |  |  |  |
|  |  |  | 1290.68 | *VQQTVQDLFGR |  |  |  |  |
|  |  |  | 1361.74 | *AQFEGIVTDLIR |  |  |  |  |
|  |  |  | 1446.76 | SDIGEVILVGGMTR |  |  |  |  |
|  |  |  | 1450.72 | *TTPSVVAFTADGER |  |  |  |  |
|  |  |  | 1462.76 | *SDIGEVILVGGMTR |  |  |  |  |
|  |  |  | 1568.77 | QAVTNPNNTFYATK |  |  |  |  |
|  |  |  | 1569.83 | LYSPSQIGAFVLMK |  |  |  |  |
|  |  |  | 1592.95 | *LLGQFTLIGIPPAPR |  |  |  |  |
|  |  |  | 1645.88 | VINEPTAAALAYGLDK |  |  |  |  |
|  |  |  | 1694.85 | *NAVITVPAYFNDSQR |  |  |  |  |
|  |  |  | 1808.90 | SQVFSTAADGQTQVEIK |  |  |  |  |
|  |  |  | 2055.96 | *STNGDTFLGGEDFDQALLR |  |  |  |  |
|  |  |  | 2158.04 | ERVEAVNMAEGIIHDTETK |  |  |  |  |
|  |  |  | 2309.21 | GVPQIEVTFDIDANGIVHVSAK |  |  |  |  |
| 601 | 6 / 25 | 141 | 706.40 | VFRER | Stomatin-like protein 2 | STML2_HUMAN | 38534.07 | 6.87 |
|  |  |  | 1514.74 | APVPGTPDSLSSGSSR |  |  |  |  |
|  |  |  | 1662.75 | DVQGTDASLDEELDR |  |  |  |  |
|  |  |  | 1675.02 | *ILEPGLNILIPVLDR |  |  |  |  |
|  |  |  | 2014.08 | *NTVVLFVPQQEAWVVER |  |  |  |  |
|  |  |  | 2346.09 | ASYGVEDPEYAVTQLAQTTMR |  |  |  |  |
| 611 | 16 / 59 | 305 | 786.47 | LELQGVK | Glyoxalase domain-containing protein 4 | GLOD4_HUMAN | 34662.27 | 5.40 |
|  |  |  | 840.44 | FYLQNR |  |  |  |  |
|  |  |  | 861.50 | ALHFVFK |  |  |  |  |
|  |  |  | 950.48 | IAFSCPQK |  |  |  |  |
|  |  |  | 1017.60 | RALHFVFK |  |  |  |  |
|  |  |  | 1053.51 | IYEKDEEK |  |  |  |  |
|  |  |  | 1057.52 | GGVDHAAAFGR |  |  |  |  |
|  |  |  | 1073.62 | VTLAVSDLQK |  |  |  |  |
|  |  |  | 1218.59 | ELPDLEDLMK |  |  |  |  |
|  |  |  | 1252.60 | ALLGYADNQCK |  |  |  |  |
|  |  |  | 1374.69 | ELPDLEDLMKR |  |  |  |  |
|  |  |  | 1514.71 | SLNYWCNLLGMK |  |  |  |  |
|  |  |  | 2068.01 | LGNDFMGITLASSQAVSNAR |  |  |  |  |
|  |  |  | 2550.28 | KLEWPLTEVAEGVFETEAPGGYK |  |  |  |  |
|  |  |  | 2758.35 | ATVQVVILADPDGHEICFVGDEAFR |  |  |  |  |
|  |  |  | 2940.30 | TMVGFGPEDDHFVAELTYNYGVGDYK |  |  |  |  |
| 629 | 9 / 52 | 309 | 759.42 | QIEELK | Ubiquitin carboxyl-terminal hydrolase isozyme L1 | UCHL1_HUMAN | 24824.34 | 5.33 |
|  |  |  | 886.49 | *LGVAGQWR |  |  |  |  |
|  |  |  | 1064.56 | LGFEDGSVLK |  |  |  |  |
|  |  |  | 1842.91 | MPFPVNHGASSEDTLLK |  |  |  |  |
|  |  |  | 1858.90 | *MPFPVNHGASSEDTLLK |  |  |  |  |
|  |  |  | 1863.90 | MQLKPMEINPEMLNK |  |  |  |  |
|  |  |  | 1967.90 | *NEAIQAAHDAVAQEGQCR |  |  |  |  |
|  |  |  | 2311.15 | QTIGNSCGTIGLIHAVANNQDK |  |  |  |  |
|  |  |  | 2519.25 | *VNFHFILFNNVDGHLYELDGR |  |  |  |  |
| 649 | 10 / 50 | 385 | 720.40 | AVIFDR | Prohibitin | PHB_HUMAN | 29804.10 | 5.57 |
|  |  |  | 1058.52 | QVAQQEAER |  |  |  |  |
|  |  |  | 1149.59 | *FDAGELITQR |  |  |  |  |
|  |  |  | 1185.66 | *DLQNVNITLR |  |  |  |  |
|  |  |  | 1396.84 | ILFRPVASQLPR |  |  |  |  |
|  |  |  | 1444.66 | *IFTSIGEDYDER |  |  |  |  |
|  |  |  | 1606.84 | KLEAAEDIAYQLSR |  |  |  |  |
|  |  |  | 1998.09 | *AAELIANSLATAGDGLIELR |  |  |  |  |
|  |  |  | 2119.14 | AATFGLILDDVSLTHLTFGK |  |  |  |  |
|  |  |  | 2371.25 | FGLALAVAGGVVNSALYNVDAGHR |  |  |  |  |
|  | 9 / 47 | 145 | 987.49 | YLTNAYSR | Chloride intracellular channel protein 4 | CLIC4_HUMAN | 28640.91 | 5.45 |
|  |  |  | 1385.68 | NSRPEANEALER |  |  |  |  |
|  |  |  | 1588.85 | EEDKEPLIELFVK |  |  |  |  |
|  |  |  | 1606.80 | NFDIPKEMTGIWR |  |  |  |  |
|  |  |  | 1681.72 | *AGSDGESIGNCPFSQR |  |  |  |  |
|  |  |  | 1707.83 | MALSMPLNGLKEEDK |  |  |  |  |
|  |  |  | 2595.17 | LDEYLNSPLPDEIDENSMEDIK |  |  |  |  |
|  |  |  | 2621.37 | KPADLQNLAPGTHPPFITFNSEVK |  |  |  |  |
|  |  |  | 2777.47 | RKPADLQNLAPGTHPPFITFNSEVK |  |  |  |  |
|  |  |  |  |  |  |  |  |  |
|  |  |  |  |  |  |  |  |  |
| 721 | 8 / 30 | 291 | 774.39 | *AHYDLR | Alpha-2-HS-glycoprotein | FETUA_HUMAN | 30221.94 | 4.53 |
|  |  |  | 813.45 | FSVVYAK |  |  |  |  |
|  |  |  | 1196.63 | HTLNQIDEVK |  |  |  |  |
|  |  |  | 1660.76 | *EHAVEGDCDFQLLK |  |  |  |  |
|  |  |  | 2081.02 | HTFMGVVSLGSPSGEVSHPR |  |  |  |  |
|  |  |  | 2097.02 | *HTFMGVVSLGSPSGEVSHPR |  |  |  |  |
|  |  |  | 2579.31 | *AQLVPLPPSTYVEFTVSGTDCVAK |  |  |  |  |
|  |  |  | 3677.82 | VWPQQPSGELFEIEIDTLETTCHVLDPTPVAR |  |  |  |  |
| 787 | 9 / 50 | 195 | 752.38 | LDAMFR | Calpain small subunit 1 | CPNS1_HUMAN | 28315.71 | 5.05 |
|  |  |  | 768.37 | LDAMFR |  |  |  |  |
|  |  |  | 781.35 | *QFDTDR |  |  |  |  |
|  |  |  | 808.44 | WQAIYK |  |  |  |  |
|  |  |  | 869.44 | LGFEEFK |  |  |  |  |
|  |  |  | 1777.80 | *THYSNIEANESEEVR |  |  |  |  |
|  |  |  | 2284.95 | YSDESGNMDFDNFISCLVR |  |  |  |  |
|  |  |  | 2447.29 | *ILGGVISAISEAAAQYNPEPPPPR |  |  |  |  |
|  |  |  | 3597.66 | GGGGGGGGGGGLGGGLGNVLGGLISGAGGGGGGGGGGGGGGGGGGGGTAMR |  |  |  |  |
| 789 | 16 / 76 | 596 | 726.45 | KPEPKK | Myosin light chain 3 | MYL3_HUMAN | 21800.87 | 5.03 |
|  |  |  | 924.43 | EVEFDASK |  |  |  |  |
|  |  |  | 995.56 | *HVLATLGER |  |  |  |  |
|  |  |  | 1044.48 | EAFMLFDR |  |  |  |  |
|  |  |  | 1249.58 | EGNGTVMGAELR |  |  |  |  |
|  |  |  | 1281.63 | *ITYGQCGDVLR |  |  |  |  |
|  |  |  | 1396.75 | *ALGQNPTQAEVLR |  |  |  |  |
|  |  |  | 1501.68 | *DTGTYEDFVEGLR |  |  |  |  |
|  |  |  | 1509.75 | *IEFTPEQIEEFK |  |  |  |  |
|  |  |  | 1524.82 | *AAPAPAPPPEPERPK |  |  |  |  |
|  |  |  | 1738.84 | VFDKEGNGTVMGAELR |  |  |  |  |
|  |  |  | 1743.82 | *NKDTGTYEDFVEGLR |  |  |  |  |
|  |  |  | 1750.93 | IKIEFTPEQIEEFK |  |  |  |  |
|  |  |  | 2015.93 | MMDFETFLPMLQHISK |  |  |  |  |
|  |  |  | 2161.95 | LMAGQEDSNGCINYEAFVK |  |  |  |  |
|  |  |  | 2535.21 | IEFTPEQIEEFKEAFMLFDR |  |  |  |  |
| 811 | 18 / 82 | 634 | 924.43 | EVEFDASK | Myosin light chain 3 | MYL3_HUMAN | 21800.87 | 5.03 |
|  |  |  | 962.47 | LTEDEVEK |  |  |  |  |
|  |  |  | 995.56 | HVLATLGER |  |  |  |  |
|  |  |  | 1028.49 | EAFMLFDR |  |  |  |  |
|  |  |  | 1041.58 | MAPKKPEPK |  |  |  |  |
|  |  |  | 1044.48 | EAFMLFDR |  |  |  |  |
|  |  |  | 1249.58 | EGNGTVMGAELR |  |  |  |  |
|  |  |  | 1281.63 | *ITYGQCGDVLR |  |  |  |  |
|  |  |  | 1396.75 | *ALGQNPTQAEVLR |  |  |  |  |
|  |  |  | 1501.68 | *DTGTYEDFVEGLR |  |  |  |  |
|  |  |  | 1509.75 | *IEFTPEQIEEFK |  |  |  |  |
|  |  |  | 1524.82 | *AAPAPAPPPEPERPK |  |  |  |  |
|  |  |  | 1738.84 | VFDKEGNGTVMGAELR |  |  |  |  |
|  |  |  | 1743.82 | *NKDTGTYEDFVEGLR |  |  |  |  |
|  |  |  | 1750.93 | IKIEFTPEQIEEFK |  |  |  |  |
|  |  |  | 2015.93 | MMDFETFLPMLQHISK |  |  |  |  |
|  |  |  | 2161.95 | LMAGQEDSNGCINYEAFVK |  |  |  |  |
|  |  |  | 2535.21 | IEFTPEQIEEFKEAFMLFDR |  |  |  |  |
| 846 | 15 / 40 | 567 | 737.42 | FVLSSGK | Calreticulin | CALR_HUMAN | 46466.37 | 4.29 |
|  |  |  | 772.41 | *GEWKPR |  |  |  |  |
|  |  |  | 975.49 | GLQTSQDAR |  |  |  |  |
|  |  |  | 992.47 | QIDNPDYK |  |  |  |  |
|  |  |  | 1019.57 | VHVIFNYK |  |  |  |  |
|  |  |  | 1047.47 | DKQDEEQR |  |  |  |  |
|  |  |  | 1147.66 | KVHVIFNYK |  |  |  |  |
|  |  |  | 1219.70 | *GQTLVVQFTVK |  |  |  |  |
|  |  |  | 1410.63 | *EQFLDGDGWTSR |  |  |  |  |
|  |  |  | 1607.77 | *FYALSASFEPFSNK |  |  |  |  |
|  |  |  | 1800.84 | *IKDPDASKPEDWDER |  |  |  |  |
|  |  |  | 2391.10 | IDNSQVESGSLEDDWDFLPPK |  |  |  |  |
|  |  |  | 2519.19 | IDNSQVESGSLEDDWDFLPPKK |  |  |  |  |
|  |  |  | 2760.26 | IDDPTDSKPEDWDKPEHIPDPDAK |  |  |  |  |
|  |  |  | 2856.39 | *CKDDEFTHLYTLIVRPDNTYEVK |  |  |  |  |
| 868 | 14 / 46 | 348 | 1086.49 | *AYTNFDAER | Annexin A2 | ANXA2_HUMAN | 38472.85 | 7.56 |
|  |  |  | 1111.55 | *QDIAFAYQR |  |  |  |  |
|  |  |  | 1222.60 | TPAQYDASELK |  |  |  |  |
|  |  |  | 1244.62 | TNQELQEINR |  |  |  |  |
|  |  |  | 1421.69 | *SLYYYIQQDTK |  |  |  |  |
|  |  |  | 1476.67 | SYSPYDMLESIR |  |  |  |  |
|  |  |  | 1542.85 | *GVDEVTIVNILTNR |  |  |  |  |
|  |  |  | 1604.76 | SYSPYDMLESIRK |  |  |  |  |
|  |  |  | 1632.84 | GTDVPKWISIMTER |  |  |  |  |
|  |  |  | 1777.86 | GLGTDEDSLIEIICSR |  |  |  |  |
|  |  |  | 1811.87 | TDLEKDIISDTSGDFR |  |  |  |  |
|  |  |  | 1844.90 | LSLEGDHSTPPSAYGSVK |  |  |  |  |
|  |  |  | 1908.88 | AEDGSVIDYELIDQDAR |  |  |  |  |
|  |  |  | 2064.98 | RAEDGSVIDYELIDQDAR |  |  |  |  |
| 891 | 12 / 83 | 452 | 762.42 | NIVAAMK | Flavin reductase | BLVRB_HUMAN | 21988.16 | 7.31 |
|  |  |  | 1141.56 | HDLGHFMLR |  |  |  |  |
|  |  |  | 1167.61 | *LQAVTDDHIR |  |  |  |  |
|  |  |  | 1493.69 | NDLSPTTVMSEGAR |  |  |  |  |
|  |  |  | 1512.87 | *TVAGQDAVIVLLGTR |  |  |  |  |
|  |  |  | 1707.88 | VVACTSAFLLWDPTK |  |  |  |  |
|  |  |  | 2157.15 | VVACTSAFLLWDPTKVPPR |  |  |  |  |
|  |  |  | 2301.94 | CLTTDEYDGHSTYPSHQYQ |  |  |  |  |
|  |  |  | 2469.31 | LPSEGPRPAHVVVGDVLQAADVDK |  |  |  |  |
|  |  |  | 2687.35 | *YVAVMPPHIGDQPLTGAYTVTLDGR |  |  |  |  |
|  |  |  | 3048.67 | *IAIFGATGQTGLTTLAQAVQAGYEVTVLVR |  |  |  |  |
|  |  |  | 3176.77 | KIAIFGATGQTGLTTLAQAVQAGYEVTVLVR |  |  |  |  |
|  |  |  |  |  |  |  |  |  |

^a^Number of matched peptides

^b^Percentage of coverage of full length protein by tryptic peptides
