# Supplementary material for: Cardiac protein changes in ischaemic and dilated cardiomyopathy: a proteomic study of human left ventricular tissue
Source: J Cell Mol Med. 2012 Sep 26;16(10):2471–86. doi: 10.1111/j.1582-4934.2012.01565.x (PMC3823441; doi:10.1111/j.1582-4934.2012.01565.x)
Supplement: Supplementary file 3 [file jcmm0016-2471-SD3.doc]

**Supplementary table 3:** Additional data on MS protein identification of ICM and DCM spots with differential expression by MALDI-MS. Peptides submitted to MS/MS. as an additional analysis that led to the same protein identification. are marked with an asterisk.

| **Spot** | **N^a^ / %^b^** | **Mascot Score** | **Peptides identified by MS** | | **Identified protein** | **Accession number** | **Expected Mw** | **Expected pI** |
| --- | --- | --- | --- | --- | --- | --- | --- | --- |
|  |  |  | **M+H** | **Sequence** |  |  |  |  |
| 18 | 11 / 41 | 260 | 705.38 | KPGMTR | Malate dehydrogenase, mitochondrial | MDHM_HUMAN | 33000.45 | 8.54 |
|  |  |  | 772.43 | NSPLVSR |  |  |  |  |
|  |  |  | 992.54 | ANTFVAELK |  |  |  |  |
|  |  |  | 1233.72 | *IFGVTTLDIVR |  |  |  |  |
|  |  |  | 1338.72 | *GCDVVVIPAGVPR |  |  |  |  |
|  |  |  | 1343.67 | FVFSLVDAMNGK |  |  |  |  |
|  |  |  | 1470.69 | AGAGSATLSMAYAGAR |  |  |  |  |
|  |  |  | 1489.74 | GYLGPEQLPDCLK |  |  |  |  |
|  |  |  | 1560.81 | *VDFPQDQLTALTGR |  |  |  |  |
|  |  |  | 1922.93 | VSSFEEKMISDAIPELK |  |  |  |  |
|  |  |  | 2365.23 | LTLYDIAHTPGVAADLSHIETK |  |  |  |  |
|  | 6 / 29 | 111 | 1023.53 | IICQGFTGK | Succinyl-CoA ligase [GDP-forming] subunit alpha, mitochondrial | SUCA_HUMAN | 32226.06 | 8.77 |
|  |  |  | 1567.86 | GGQTHLGLPVFNTVK |  |  |  |  |
|  |  |  | 1694.82 | *QGTFHSQQALEYGTK |  |  |  |  |
|  |  |  | 1724.84 | LIGPNCPGVINPGECK |  |  |  |  |
|  |  |  | 2431.30 | QHNSGPNSKPVVSFIAGLTAPPGR |  |  |  |  |
|  |  |  | 2437.30 | ISALQSAGVVVSMSPAQLGTTIYK |  |  |  |  |
| 70 | 18 / 42 | 272 | 787.42 | QAHLYR | Pyruvate kinase isozymes M1/M2 | KPYM_HUMAN | 57805.70 | 7.95 |
|  |  |  | 840.52 | APIIAVTR |  |  |  |  |
|  |  |  | 884.45 | MQHLIAR |  |  |  |  |
|  |  |  | 933.52 | GIFPVLCK |  |  |  |  |
|  |  |  | 953.48 | IENHEGVR |  |  |  |  |
|  |  |  | 1019.52 | *GDYPLEAVR |  |  |  |  |
|  |  |  | 1197.65 | LDIDSPPITAR |  |  |  |  |
|  |  |  | 1359.73 | NTGIICTIGPASR |  |  |  |  |
|  |  |  | 1462.81 | IYVDDGLISLQVK |  |  |  |  |
|  |  |  | 1636.89 | GVNLPGAAVDLPAVSEK |  |  |  |  |
|  |  |  | 1642.78 | *DPVQEAWAEDVDLR |  |  |  |  |
|  |  |  | 1681.82 | FDEILEASDGIMVAR |  |  |  |  |
|  |  |  | 1779.89 | GADFLVTEVENGGSLGSK |  |  |  |  |
|  |  |  | 1837.92 | RFDEILEASDGIMVAR |  |  |  |  |
|  |  |  | 1875.90 | *FGVEQDVDMVFASFIR |  |  |  |  |
|  |  |  | 1883.91 | LNFSHGTHEYHAETIK |  |  |  |  |
|  |  |  | 2279.11 | GDVVIVLTGWRPGSGFTNTMR |  |  |  |  |
|  |  |  | 2465.31 | TATESFASDPILYRPVAVALDTK |  |  |  |  |
| 78 | 13 / 31 | 181 | 722.36 | DLDGFR | UTP-glucose-1-phosphatase uridylyltransferase | UGPA_HUMAN | 56809.06 | 8.15 |
|  |  |  | 793.42 | VQDYLR |  |  |  |  |
|  |  |  | 1028.52 | *IYTFNQSR |  |  |  |  |
|  |  |  | 1265.69 | GTVIIIANHGDR |  |  |  |  |
|  |  |  | 1332.69 | *SFENSLGINVPR |  |  |  |  |
|  |  |  | 1575.78 | SFENSLGINVPRSR |  |  |  |  |
|  |  |  | 1682.78 | AMSQDGASQFQEVIR |  |  |  |  |
|  |  |  | 1699.86 | *IQRPPEDSIQPYEK |  |  |  |  |
|  |  |  | 1983.10 | TLDGGLNVIQLETAVGAAIK |  |  |  |  |
|  |  |  | 2156.09 | NENTFLDLTVQQIEHLNK |  |  |  |  |
|  |  |  | 2545.14 | TYNTDVPLVLMNSFNTDEDTKK |  |  |  |  |
|  |  |  | 2561.15 | TYNTDVPLVLMNSFNTDEDTKK |  |  |  |  |
|  |  |  | 2720.31 | TTSDLLLVMSNLYSLNAGSLTMSEK |  |  |  |  |
| 110 | 8 / 33 | 339 | 793.36 | *FNENNR | Galectin-3 | LEG3_HUMAN | 26021.14 | 8.60 |
|  |  |  | 862.48 | *IALDFQR |  |  |  |  |
|  |  |  | 874.42 | LDNNWGR |  |  |  |  |
|  |  |  | 1273.61 | *GNDVAFHFNPR |  |  |  |  |
|  |  |  | 1324.73 | *IQVLVEPDHFK |  |  |  |  |
|  |  |  | 1497.78 | *QSVFPFESGKPFK |  |  |  |  |
|  |  |  | 1649.87 | *VAVNDAHLLQYNHR |  |  |  |  |
|  |  |  | 1656.94 | MLITILGTVKPNANR |  |  |  |  |
| 115 | 6 / 30 | 72 | 1135.59 | *NGFLLDGFPR | Adenylate kinase 2, mitochondrial | KAD2_HUMAN | 26346.55 | 7.85 |
|  |  |  | 1193.62 | AMVASGSELGKK |  |  |  |  |
|  |  |  | 1817.01 | LDSVIEFSIPDSLLIR |  |  |  |  |
|  |  |  | 1997.01 | LQAYHTQTTPLIEYYR |  |  |  |  |
|  |  |  | 2022.92 | LAENFCVCHLATGDMLR |  |  |  |  |
|  |  |  | 2074.14 | EKLDSVIEFSIPDSLLIR |  |  |  |  |
| 121 | 11 / 34 | 310 | 866.48 | *FFSPLQK | 2,4-dienoyl-CoA reductase, mitochondrial | DECR_HUMAN | 32149.96 | 8.79 |
|  |  |  | 1097.55 | VHAIQCDVR |  |  |  |  |
|  |  |  | 1112.65 | *FNVIQPGPIK |  |  |  |  |
|  |  |  | 1177.65 | VAFITGGGTGLGK |  |  |  |  |
|  |  |  | 1189.63 | AMLPPNSFQGK |  |  |  |  |
|  |  |  | 1205.60 | AMLPPNSFQGK |  |  |  |  |
|  |  |  | 1431.72 | *EQWDTIEELIR |  |  |  |  |
|  |  |  | 1604.77 | DPDMVQNTVSELIK |  |  |  |  |
|  |  |  | 1896.91 | *FDGGEEVLISGEFNDLR |  |  |  |  |
|  |  |  | 2025.00 | FDGGEEVLISGEFNDLRK |  |  |  |  |
|  |  |  | 2291.20 | *VAGHPNIVINNAAGNFISPTER |  |  |  |  |
| 165 | 5 / 34 | 261 | 707.37 | RFEQK | Nucleoside diphosphate kinase B | NDKB_HUMAN | 17166.84 | 8.55 |
|  |  |  | 1051.50 | *GDFCIQVGR |  |  |  |  |
|  |  |  | 1175.66 | *DRPFFPGLVK |  |  |  |  |
|  |  |  | 1344.77 | *TFIAIKPDGVQR |  |  |  |  |
|  |  |  | 1801.91 | *VMLGETNPADSKPGTIR |  |  |  |  |
| 192 | 13 / 34 | 171 | 704.40 | GVPLYR | Alpha-enolase | ENOA_HUMAN | 47037.77 | 6.99 |
|  |  |  | 766.37 | *EIFDSR |  |  |  |  |
|  |  |  | 806.45 | YNQLLR |  |  |  |  |
|  |  |  | 869.44 | YMGKGVSK |  |  |  |  |
|  |  |  | 899.53 | TIAPALVSK |  |  |  |  |
|  |  |  | 1425.74 | YISPDQLADLYK |  |  |  |  |
|  |  |  | 1519.80 | FGANAILGVSLAVCK |  |  |  |  |
|  |  |  | 1541.74 | LAQANGWGVMVSHR |  |  |  |  |
|  |  |  | 1556.77 | *VVIGMDVAASEFFR |  |  |  |  |
|  |  |  | 1804.94 | *AAVPSGASTGIYEALELR |  |  |  |  |
|  |  |  | 1828.92 | VVIGMDVAASEFFRSGK |  |  |  |  |
|  |  |  | 1939.96 | LAMQEFMILPVGAANFR |  |  |  |  |
|  |  |  | 2353.13 | SGETEDTFIADLVVGLCTGQIK |  |  |  |  |
| 197 | 14 / 31 | 238 | 707.38 | QAVAYR | ATP synthase subunit alpha, mitochondrial | ATPA_HUMAN | 55209.32 | 8.28 |
|  |  |  | 716.46 | LTELLK |  |  |  |  |
|  |  |  | 723.45 | APGIIPR |  |  |  |  |
|  |  |  | 815.47 | GPIGSKTR |  |  |  |  |
|  |  |  | 892.49 | LELAQYR |  |  |  |  |
|  |  |  | 1026.59 | AVDSLVPIGR |  |  |  |  |
|  |  |  | 1553.74 | *EAYPGDVFYLHSR |  |  |  |  |
|  |  |  | 1564.86 | QAVAYRQMSLLLR |  |  |  |  |
|  |  |  | 1575.79 | ILGADTSVDLEETGR |  |  |  |  |
|  |  |  | 1624.89 | *TGAIVDVPVGEELLGR |  |  |  |  |
|  |  |  | 1683.79 | NVQAEEMVEFSSGLK |  |  |  |  |
|  |  |  | 2325.15 | QGQYSPMAIEEQVAVIYAGVR |  |  |  |  |
|  |  |  | 2338.17 | EVAAFAQFGSDLDAATQQLLSR |  |  |  |  |
|  |  |  | 2367.26 | FENAFLSHVVSQHQALLGTIR |  |  |  |  |
| 228 | 18 / 50 | 759 | 776.44 | *AGNVIFR | Succinyl-CoA:3-ketoacid-coenzyme A transferase 1, mitochondrial | SCOT1_HUMAN | 52089.89 | 6.00 |
|  |  |  | 793.41 | AVFDVDK |  |  |  |  |
|  |  |  | 944.48 | SAKPGDDVR |  |  |  |  |
|  |  |  | 1039.47 | NFNLPMCK |  |  |  |  |
|  |  |  | 1077.51 | LMPMQQIAN |  |  |  |  |
|  |  |  | 1100.61 | DGSVAIASKPR |  |  |  |  |
|  |  |  | 1168.59 | FYTDPVEAVK |  |  |  |  |
|  |  |  | 1380.66 | YGDLANWMIPGK |  |  |  |  |
|  |  |  | 1581.78 | MVKGMGGAMDLVSSAK |  |  |  |  |
|  |  |  | 1617.72 | MVSSYVGENAEFER |  |  |  |  |
|  |  |  | 1633.72 | *MVSSYVGENAEFER |  |  |  |  |
|  |  |  | 1675.81 | GGHVDLTMLGAMQVSK |  |  |  |  |
|  |  |  | 2101.14 | *GLTAVSNNAGVDNFGLGLLLR |  |  |  |  |
|  |  |  | 2233.13 | *QYLSGELEVELTPQGTLAER |  |  |  |  |
|  |  |  | 2378.20 | *EFNGQHFILEEAITGDFALVK |  |  |  |  |
|  |  |  | 2421.16 | ETVTILPGASFFSSDESFAMIR |  |  |  |  |
|  |  |  | 2595.31 | *AGGAGVPAFYTPTGYGTLVQEGGSPIK |  |  |  |  |
|  |  |  | 3560.83 | AAETTVVEVEEIVDIGAFAPEDIHIPQIYVHR |  |  |  |  |
| 239 | 5 / 29 | 235 | 793.38 | REGMER | Malate dehydrogenase, cytoplasmic | MDHC_HUMAN | 36294.93 | 6.89 |
|  |  |  | 1026.48 | *ENFSCLTR |  |  |  |  |
|  |  |  | 1164.61 | *GEFVTTVQQR |  |  |  |  |
|  |  |  | 1393.72 | *FVEGLPINDFSR |  |  |  |  |
|  |  |  | 1401.74 | DLDVAILVGSMPR |  |  |  |  |
| 283 | 3 / 46 | 64 | 1711.76 | SYSCQVTHEGSTVEK | Ig lambda-3 chain C regions | LAC3_HUMAN | 10167.32 | 7.22 |
|  |  |  | 1743.85 | YAASSYLSLTPEQWK |  |  |  |  |
|  |  |  | 1986.01 | *AAPSVTLFPPSSEELQANK |  |  |  |  |
| 300 | 5 / 23 | 99 | 824.46 | GFGFGLVK | Voltage-dependent anion-selective channel protein 2 | VDAC2_HUMAN | 31435.31 | 7.66 |
|  |  |  | 914.50 | VGLALELEA |  |  |  |  |
|  |  |  | 940.46 | *NNFAVGYR |  |  |  |  |
|  |  |  | 2103.17 | *VNNSSLIGVGYTQTLRPGVK |  |  |  |  |
|  |  |  | 2528.20 | TGDFQLHTNVNDGTEFGGSIYQK |  |  |  |  |
| 304 | 5 / 36 | 100 | 841.49 | VTVLGQPK | Immunoglobulin lambda-like polypeptide 5 | IGLL5_HUMAN | 19278.73 | 9.03 |
|  |  |  | 1711.77 | SYSCQVTHEGSTVEK |  |  |  |  |
|  |  |  | 1743.86 | YAASSYLSLTPEQWK |  |  |  |  |
|  |  |  | 2043.02 | *ANPTVTLFPPSSEELQANK |  |  |  |  |
|  |  |  | 2296.18 | MRPKTGQVGCETPEELGPGPR |  |  |  |  |
| 318 | 8 / 51 | 169 | 889.47 | NVLTESAR | ES1 protein homolog, mitochondrial | ES1_HUMAN | 24016.57 | 6.63 |
|  |  |  | 946.42 | GQPSEGESR |  |  |  |  |
|  |  |  | 1153.58 | EVVEAHVDQK |  |  |  |  |
|  |  |  | 1206.62 | WPYAGTAEAIK |  |  |  |  |
|  |  |  | 2405.19 | GGAEVQIFAPDVPQMHVIDHTK |  |  |  |  |
|  |  |  | 2442.24 | *ITDLANLSAANHDAAIFPGGFGAAK |  |  |  |  |
|  |  |  | 2821.35 | VVTTPAFMCETALHYIHDGIGAMVR |  |  |  |  |
|  |  |  | 2966.54 | VALVLSGCGVYDGTEIHEASAILVHLSR |  |  |  |  |
| 326 | 3 / 48 | 90 | 1797.89 | *SGTASVVCLLNNFYPR | Ig kappa chain C region | IGKC_HUMAN | 10820.97 | 5.58 |
|  |  |  | 1875.92 | VYACEVTHQGLSSPVTK |  |  |  |  |
|  |  |  | 1946.02 | *TVAAPSVFIFPPSDEQLK |  |  |  |  |
| 335 | 9 / 65 | 392 | 769.35 | *VFNDMK | Cofilin-2 | COF2_HUMAN | 18605.42 | 7.88 |
|  |  |  | 929.52 | QIIVEEAK |  |  |  |  |
|  |  |  | 1000.53 | *LLPLNDCR |  |  |  |  |
|  |  |  | 1323.68 | *AVLFCLSDDKR |  |  |  |  |
|  |  |  | 1337.63 | *YALYDATYETK |  |  |  |  |
|  |  |  | 1381.78 | LGGNVVVSLEGKPL |  |  |  |  |
|  |  |  | 1453.71 | *HEWQVNGLDDIK |  |  |  |  |
|  |  |  | 1724.83 | HEWQVNGLDDIKDR |  |  |  |  |
|  |  |  | 1990.06 | KEDLVFIFWAPESAPLK |  |  |  |  |
| 344 | 5 / 42 | 149 | 748.43 | *ALELFR | Myoglobin | MYG_HUMAN | 17052.61 | 7.29 |
|  |  |  | 1350.78 | HGATVLTALGGILK |  |  |  |  |
|  |  |  | 1531.68 | HPGDFGADAQGAMNK |  |  |  |  |
|  |  |  | 1632.88 | *VEADIPGHGQEVLIR |  |  |  |  |
|  |  |  | 1970.02 | YLEFISECIIQVLQSK |  |  |  |  |
| 447 | 6 / 26 | 221 | 1214.62 | LGTQPYFFNK | Metaxin-2 | MTX2_HUMAN | 29763.19 | 5.90 |
|  |  |  | 1236.56 | IEQHYFEDR |  |  |  |  |
|  |  |  | 1257.60 | NYSNLLAFCR |  |  |  |  |
|  |  |  | 1392.67 | *RIEQHYFEDR |  |  |  |  |
|  |  |  | 2278.04 | *TLDQVLEDVDQCCQALSQR |  |  |  |  |
|  |  |  | 2406.35 | *VPFIHVGNQVVSELGPIVQFVK |  |  |  |  |
| 460 | 3 / 50 | 122 | 1797.90 | *SGTASVVCLLNNFYPR | Ig kappa chain C region | IGKC_HUMAN | 11608.86 | 5.58 |
|  |  |  | 1875.93 | *VYACEVTHQGLSSPVTK |  |  |  |  |
|  |  |  | 2135.97 | VDNALQSGNSQESVTEQDSK |  |  |  |  |
| 469 | 8 / 28 | 273 | 837.46 | ISWYLR | Delta(3,5)-Delta(2,4)-dienoyl-CoA isomerase. mitochondrial | ECH1_HUMAN | 32205.87 | 5.99 |
|  |  |  | 864.49 | VNLLYSR |  |  |  |  |
|  |  |  | 1298.64 | *YQETFNVIER |  |  |  |  |
|  |  |  | 1376.63 | YCAQDAFFQVK |  |  |  |  |
|  |  |  | 1454.84 | HVLHVQLNRPNK |  |  |  |  |
|  |  |  | 1542.81 | *EVDVGLAADVGTLQR |  |  |  |  |
|  |  |  | 1731.93 | EVMLDAALALAAEISSK |  |  |  |  |
|  |  |  | 1731.93 | *VIGNQSLVNELAFTAR |  |  |  |  |
| 490 | 4 / 13 | 103 | 763.41 | MLSVASR | Cytochrome b-c1 complex subunit Rieske, mitochondrial | UCRI_HUMAN | 49128.60 | 5.43 |
|  |  |  | 816.51 | GKPLFVR |  |  |  |  |
|  |  |  | 1012.47 | *VPDFSEYR |  |  |  |  |
|  |  |  | 1614.83 | *EIEQEAAVELSQLR |  |  |  |  |
| 496 | 3 / 19 | 71 | 1051.51 | GDFCIQVGR | Nucleoside diphosphate kinase A | NDKA_HUMAN | 17017.53 | 5.82 |
|  |  |  | 1149.63 | *DRPFFAGLVK |  |  |  |  |
|  |  |  | 1197.55 | FMQASEDLLK |  |  |  |  |
| 553 | 7 / 15 | 70 | 795.46 | IIAPPER | Actin, alpha cardiac muscle 1 | ACTC_HUMAN | 41784.64 | 5.23 |
|  |  |  | 976.46 | AGFAGDDAPR |  |  |  |  |
|  |  |  | 1130.54 | *GYSFVTTAER |  |  |  |  |
|  |  |  | 1130.54 | GYSFVTTAER |  |  |  |  |
|  |  |  | 1198.68 | AVFPSIVGRPR |  |  |  |  |
|  |  |  | 2278.07 | DLYANNVMSGGTTMYPGIADR |  |  |  |  |
|  |  |  | 2297.18 | VAPEEHPTLLTEAPLNPKANR |  |  |  |  |
| 601 | 6 / 25 | 141 | 706.40 | VFRER | Stomatin-like protein 2 | STML2_HUMAN | 38534.07 | 6.87 |
|  |  |  | 1514.74 | APVPGTPDSLSSGSSR |  |  |  |  |
|  |  |  | 1662.75 | DVQGTDASLDEELDR |  |  |  |  |
|  |  |  | 1675.02 | *ILEPGLNILIPVLDR |  |  |  |  |
|  |  |  | 2014.08 | *NTVVLFVPQQEAWVVER |  |  |  |  |
|  |  |  | 2346.09 | ASYGVEDPEYAVTQLAQTTMR |  |  |  |  |
| 611 | 16 / 59 | 305 | 786.47 | LELQGVK | Glyoxalase domain-containing protein 4 | GLOD4_HUMAN | 34662.27 | 5.40 |
|  |  |  | 840.44 | FYLQNR |  |  |  |  |
|  |  |  | 861.50 | ALHFVFK |  |  |  |  |
|  |  |  | 950.48 | IAFSCPQK |  |  |  |  |
|  |  |  | 1017.60 | RALHFVFK |  |  |  |  |
|  |  |  | 1053.51 | IYEKDEEK |  |  |  |  |
|  |  |  | 1057.52 | GGVDHAAAFGR |  |  |  |  |
|  |  |  | 1073.62 | VTLAVSDLQK |  |  |  |  |
|  |  |  | 1218.59 | ELPDLEDLMK |  |  |  |  |
|  |  |  | 1252.60 | ALLGYADNQCK |  |  |  |  |
|  |  |  | 1374.69 | ELPDLEDLMKR |  |  |  |  |
|  |  |  | 1514.71 | SLNYWCNLLGMK |  |  |  |  |
|  |  |  | 2068.01 | LGNDFMGITLASSQAVSNAR |  |  |  |  |
|  |  |  | 2550.28 | KLEWPLTEVAEGVFETEAPGGYK |  |  |  |  |
|  |  |  | 2758.35 | ATVQVVILADPDGHEICFVGDEAFR |  |  |  |  |
|  |  |  | 2940.30 | TMVGFGPEDDHFVAELTYNYGVGDYK |  |  |  |  |
| 640 | 8 / 51 | 217 | 831.51 | VPFSLLR | Heat shock protein beta-1 | HSPB1_HUMAN | 22782.52 | 5.98 |
|  |  |  | 960.43 | DWYPHSR |  |  |  |  |
|  |  |  | 961.45 | GPSWDPFR |  |  |  |  |
|  |  |  | 1104.51 | QDEHGYISR |  |  |  |  |
|  |  |  | 1163.62 | *LFDQAFGLPR |  |  |  |  |
|  |  |  | 1783.92 | VSLDVNHFAPDELTVK |  |  |  |  |
|  |  |  | 1905.99 | *LATQSNEITIPVTFESR |  |  |  |  |
|  |  |  | 3242.65 | KYTLPPGVDPTQVSSSLSPEGTLTVEAPMPK |  |  |  |  |
| 739 | 18 / 50 | 475 | 750.40 | FLEDVK | Alpha-1-antitrypsin | A1AT_HUMAN | 44324.55 | 5.37 |
|  |  |  | 795.41 | SPLFMGK |  |  |  |  |
|  |  |  | 922.43 | FLENEDR |  |  |  |  |
|  |  |  | 1078.53 | *FLENEDRR |  |  |  |  |
|  |  |  | 1090.57 | WERPFEVK |  |  |  |  |
|  |  |  | 1263.60 | LGMFNIQHCK |  |  |  |  |
|  |  |  | 1275.68 | *GKWERPFEVK |  |  |  |  |
|  |  |  | 1576.84 | DTVFALVNYIFFK |  |  |  |  |
|  |  |  | 1641.86 | *ITPNLAEFAFSLYR |  |  |  |  |
|  |  |  | 1779.77 | TDTSHHDQDHPTFNK |  |  |  |  |
|  |  |  | 1803.96 | LQHLENELTHDIITK |  |  |  |  |
|  |  |  | 1833.92 | VFSNGADLSGVTEEAPLK |  |  |  |  |
|  |  |  | 1871.97 | *FNKPFVFLMIEQNTK |  |  |  |  |
|  |  |  | 1891.86 | *DTEEEDFHVDQVTTVK |  |  |  |  |
|  |  |  | 2057.95 | LYHSEAFTVNFGDTEEAK |  |  |  |  |
|  |  |  | 2186.04 | LYHSEAFTVNFGDTEEAKK |  |  |  |  |
|  |  |  | 2291.13 | GTEAAGAMFLEAIPMSIPPEVK |  |  |  |  |
|  |  |  | 2574.34 | *TLNQPDSQLQLTTGNGLFLSEGLK |  |  |  |  |
| 781 | 6 / 16 | 101 | 722.34 | *AEFAER | Tropomyosin alpha-4 | TPM4_HUMAN | 28390.62 | 4.67 |
|  |  |  | 1170.68 | *LVILEGELER |  |  |  |  |
|  |  |  | 1243.66 | *IQLVEEELDR |  |  |  |  |
|  |  |  | 1298.77 | KLVILEGELER |  |  |  |  |
|  |  |  | 1614.78 | IQALQQQADEAEDR |  |  |  |  |
|  |  |  | 1742.87 | KIQALQQQADEAEDR |  |  |  |  |
| 788 | 10 / 40 | 390 | 734.35 | DDESLR | Rho GDP-dissociation inhibitor 1 | GDIR1_HUMAN | 23075.92 | 5.01 |
|  |  |  | 752.35 | *EGVEYR |  |  |  |  |
|  |  |  | 980.50 | YIQHTYR |  |  |  |  |
|  |  |  | 1202.62 | SIQEIQELDK |  |  |  |  |
|  |  |  | 1261.55 | *TDYMVGSYGPR |  |  |  |  |
|  |  |  | 1617.75 | *IDKTDYMVGSYGPR |  |  |  |  |
|  |  |  | 1751.82 | *AEEYEFLTPVEEAPK |  |  |  |  |
|  |  |  | 1917.93 | *SIQEIQELDKDDESLR |  |  |  |  |
|  |  |  | 2296.13 | AEEYEFLTPVEEAPKGMLAR |  |  |  |  |
|  |  |  | 2364.10 | FTDDDKTDHLSWEWNLTIK |  |  |  |  |
|  | 5 / 28 | 117 | 984.48 | EAFSLFDR | Myosin light chain 4 | MYL4_HUMAN | 21433.41 | 4.98 |
|  |  |  | 1281.62 | ITYGQCGDVLR |  |  |  |  |
|  |  |  | 1382.74 | ALGQNPTNAEVLR |  |  |  |  |
|  |  |  | 1455.69 | IDFTADQIEEFK |  |  |  |  |
|  |  |  | 1542.70 | *EQGTYEDFVEGLR |  |  |  |  |
| 811 | 18 / 82 | 634 | 924.43 | EVEFDASK | Myosin light chain 3 | MYL3_HUMAN | 21800.87 | 5.03 |
|  |  |  | 962.47 | LTEDEVEK |  |  |  |  |
|  |  |  | 995.56 | HVLATLGER |  |  |  |  |
|  |  |  | 1028.49 | EAFMLFDR |  |  |  |  |
|  |  |  | 1041.58 | MAPKKPEPK |  |  |  |  |
|  |  |  | 1044.48 | EAFMLFDR |  |  |  |  |
|  |  |  | 1249.58 | EGNGTVMGAELR |  |  |  |  |
|  |  |  | 1281.63 | *ITYGQCGDVLR |  |  |  |  |
|  |  |  | 1396.75 | *ALGQNPTQAEVLR |  |  |  |  |
|  |  |  | 1501.68 | *DTGTYEDFVEGLR |  |  |  |  |
|  |  |  | 1509.75 | *IEFTPEQIEEFK |  |  |  |  |
|  |  |  | 1524.82 | *AAPAPAPPPEPERPK |  |  |  |  |
|  |  |  | 1738.84 | VFDKEGNGTVMGAELR |  |  |  |  |
|  |  |  | 1743.82 | *NKDTGTYEDFVEGLR |  |  |  |  |
|  |  |  | 1750.93 | IKIEFTPEQIEEFK |  |  |  |  |
|  |  |  | 2015.93 | MMDFETFLPMLQHISK |  |  |  |  |
|  |  |  | 2161.95 | LMAGQEDSNGCINYEAFVK |  |  |  |  |
|  |  |  | 2535.21 | IEFTPEQIEEFKEAFMLFDR |  |  |  |  |
| 812 | 6 / 26 | 62 | 816.47 | IAWALSR | Lactoylglutathione lyase | LGUL_HUMAN | 20646.52 | 5.12 |
|  |  |  | 901.44 | *SLDFYTR |  |  |  |  |
|  |  |  | 977.54 | RFEELGVK |  |  |  |  |
|  |  |  | 1280.67 | *DFLLQQTMLR |  |  |  |  |
|  |  |  | 1395.69 | FSLYFLAYEDK |  |  |  |  |
|  |  |  | 1962.94 | FSLYFLAYEDKNDIPK |  |  |  |  |
| 890 | 10 / 37 | 222 | 909.42 | AANWYER | NADH dehydrogenase [ubiquinone] iron-sulfur protein 3, mitochondrial | NDUS3_HUMAN | 26414.92 | 5.48 |
|  |  |  | 1295.66 | DFPLSGYVELR |  |  |  |  |
|  |  |  | 1366.77 | FEIVYNLLSLR |  |  |  |  |
|  |  |  | 1385.76 | SLVDLTAVDVPTR |  |  |  |  |
|  |  |  | 1486.79 | *VVAEPVELAQEFR |  |  |  |  |
|  |  |  | 1512.79 | ESAGADTRPTVRPR |  |  |  |  |
|  |  |  | 1551.76 | *ILTDYGFEGHPFR |  |  |  |  |
|  |  |  | 1707.86 | RILTDYGFEGHPFR |  |  |  |  |
|  |  |  | 1740.84 | FDLNSPWEAFPVYR |  |  |  |  |
|  |  |  | 1868.93 | KFDLNSPWEAFPVYR |  |  |  |  |
| 1198 | 11 / 26 | 98 | 707.37 | EQFRK | Heterogeneous nuclear ribonucleoproteins A2/B1 | ROA2_HUMAN | 37429.70 | 8.97 |
|  |  |  | 727.41 | VVEPKR |  |  |  |  |
|  |  |  | 733.44 | LFVGGIK |  |  |  |  |
|  |  |  | 836.42 | EKEQFR |  |  |  |  |
|  |  |  | 1050.44 | DYFEEYGK |  |  |  |  |
|  |  |  | 1087.49 | NYYEQWGK |  |  |  |  |
|  |  |  | 1188.64 | IDTIEIITDR |  |  |  |  |
|  |  |  | 1237.55 | QEMQEVQSSR |  |  |  |  |
|  |  |  | 1338.81 | EESGKPGAHVTVK |  |  |  |  |
|  |  |  | 1695.76 | GFGFVTFDDHDPVDK |  |  |  |  |
|  |  |  | 1798.92 | *LFIGGLSFETTEESLR |  |  |  |  |
|  |  |  |  |  |  |  |  |  |

^a^Number of matched peptides

^b^Percentage of coverage of full length protein by tryptic peptides
